# Sim3C: simulation of Hi-C and Meta3C proximity ligation sequencing technologies

**DOI:** 10.1093/gigascience/gix103

**Published:** 2017-11-15

**Authors:** Matthew Z DeMaere, Aaron E Darling

**Affiliations:** The ithree institute, University of Technology Sydney, PO Box 123, Broadway, NSW 2077, Australia

**Keywords:** Hi-C, Meta3C, 3C, DNA sequencing, simulation, metagenomics

## Abstract

**Background:**

Chromosome conformation capture (3C) and Hi-C DNA sequencing methods have rapidly advanced our understanding of the spatial organization of genomes and metagenomes. Many variants of these protocols have been developed, each with their own strengths. Currently there is no systematic means for simulating sequence data from this family of sequencing protocols, potentially hindering the advancement of algorithms to exploit this new datatype.

**Findings:**

We describe a computational simulator that, given simple parameters and reference genome sequences, will simulate Hi-C sequencing on those sequences. The simulator models the basic spatial structure in genomes that is commonly observed in Hi-C and 3C datasets, including the distance-decay relationship in proximity ligation, differences in the frequency of interaction within and across chromosomes, and the structure imposed by cells. A means to model the 3D structure of randomly generated topologically associating domains is provided. The simulator considers several sources of error common to 3C and Hi-C library preparation and sequencing methods, including spurious proximity ligation events and sequencing error.

**Conclusions:**

We have introduced the first comprehensive simulator for 3C and Hi-C sequencing protocols. We expect the simulator to have use in testing of Hi-C data analysis algorithms, as well as more general value for experimental design, where questions such as the required depth of sequencing, enzyme choice, and other decisions can be made in advance in order to ensure adequate statistical power with respect to experimental hypothesis testing.

## Findings

### Software testing

To the casual observer, formal software testing is often thought to begin and end with the validation of fine-grained behavioural (functional) aspects; such as the correct execution of individual methods. In day-to-day use, however, what can matter most to end users are broader system attributes such as speed, scalability, reproducibility, and ease of use. To ensure that a project offers maximum value, a thorough testing process would collectively examine all aspects.

For inferential software within scientific fields, the system-level attributes of precision and accuracy are of primary interest, and their quantification is best accomplished by comparison with a known truth (gold standard). Therefore, any testing methodology capable of providing an *a priori* gold standard, particularly without estimation, improves this facet of testing significantly.

Purpose-built bioinformatics software ultimately acts on experimentally collected observations. The inherent noise and variation that comes with experimental data means that achieving testing thoroughness is a great challenge. Ready access to sufficient data sources is a fundamental necessity for adequate software testing.

For established experimental methods, public data archives are a first choice for the necessary testing data. When high-quality metadata are available, testing driven by real data becomes possible. However, even when sufficient depth and description of data are available, difficulty can remain in matching desired test data characteristics to what actually exists in 1 or several public datasets. Further, fine-grained whole-corpus querying of metadata on remote data archives is not always possible, frequently making the up-front job of data selection a difficult task. Once selected, obtaining said real data can be time-consuming or even infeasible in locations with lower network speeds and/or high bandwidth costs. In advancing fields such as DNA sequencing, new experimental datatypes can appear for which the public data archives contain only a handful of examples, and few researchers would have the time and financial resources to commit to experimental generation of new data purely for software testing.

Though performance on real data is the ultimate arbiter of analytical value, advantaged by explicit control over its characteristics, a faithful simulation of real data can act as a valuable proxy. Simulation-driven development and testing has proven to be a highly cost-effective and time-efficient approach. It offers the possibility to explore a near continuum of data characteristics, subjecting software to an otherwise unavailable degree of testing thoroughness. Certainty and control make attaining the twin objectives of rigorous testing and an *a priori* gold standard straightforward. This enables us not only to be more certain about when we have failed, but also to extrapolate this process to infer the limits of success within the experimental parameter space.

Tools for simulating DNA sequencing reads have existed from the very early days of genomics, beginning with the many anonymous implementations of simple DNA shearing algorithms, up to the most recent highly detailed empirical model simulators [1–4]. From read simulation in isolation, field advancements such as metagenomics have been accompanied soon after by simulators reflecting their specific data characteristics and evolving experimental methodology [5–7].

We introduce Sim3C, a software package designed to simulate data generated by Hi-C and other 3C-based proximity ligation (PL) sequencing protocols. The software includes flexible support for a range of sequencing project scenarios and choice of three 3C methods (Hi-C, Meta3C, DNase Hi-C). The resulting output (paired-end FastQ) is easily assimilated into existing analysis workflows. It is our intention that Sim3C provide the Hi-C/3C research community with a means to further validate existing software projects, support new experimental or analysis development initiatives, and serve as a platform for exploration, such as the comparative analysis of clustering algorithms [8].

### 3C sequencing

3C-based sequencing protocols, including Hi-C, 4C-seq, and Meta3C, have great potential to address questions directed at the spatial organization of DNA in samples ranging from eukaryotic tissue to single cells to microbial communities. The growing use of these protocols creates a legitimate need for a simulator capable of generating data with relevant characteristics.

Chromosome conformation capture (3C) was originally designed as a polymerase chain reaction–based assay to measure interactions among a small number of defined regions of eukaryotic chromosomes [9]. In 2009, Lieberman-Aiden [10] reported an extension of the protocol to high-throughput sequencing, enabling the global spatial arrangement of chromosomes to be reconstructed at unprecedented resolution. All 3C protocols depend on an initial formalin fixation step, which crosslinks proteins bound to DNA *in vivo*. Subsequently cells are lysed and the DNA:protein complexes are sheared enzymatically and/or physically to create free ends in the bound DNA strands. These free ends are then subjected to a proximity ligation reaction, in which ligation of free ends preferentially occurs among DNA strands cobound in a protein complex. The DNA:protein crosslinks are then reversed, the DNA is purified, and an Illumina-compatible sequencing library is constructed. In Hi-C protocols, the proximity ligation junctions can then be further purified in the sequencing library.

3C-derived methods have found several applications beyond their initial use to reconstruct 3D chromosome structure. For example, it has been shown that 3C-derived data provide a valuable signal for genome scaffolding [11,12], as well as a signal that can support genome-wide haplotype phasing [13,14]. 3C-derived data have also proven valuable for metagenomics, where initial studies on mock communities demonstrated that highly accurate genome reconstruction in mixed microbial communities could be facilitated by proximity ligation sequence data [15–17]. Subsequent application to naturally occurring microbial communities has also suggested that bacteriophage can be linked to their hosts with this data type [18].

In the remainder of this manuscript, we describe the Sim3C software and demonstrate how it can be used to simulate data for various 3C-derived experiments.

### Experiment scenarios

**Figure 1:**
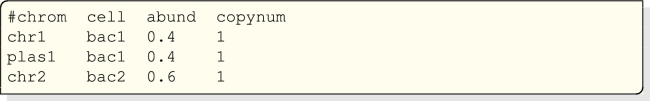
A mock 2 genome community. For demonstration purposes, we assume that the plasmid (plas1) is present in 4 copies and that there is a 0.4/0.6 relative abundance split between the 2 organisms (bac1, bac2) in the community.

**Figure 2:**
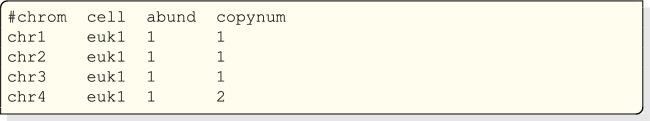
A mock 4 chromosome genome. Cellular abundance is a constant across the profile, while chr4 exists in 2 copies. Note that relative abundances specified in a profile are not required to sum to 1, but are normalized internally.

Beyond simple monochromosomal genome sequencing experiments, Sim3C offers support for the more complex scenarios of multi-chromosomal genomes and metagenomes. A scenario is defined by way of a community profile, assigning a copy-number and containing genome to each chromosome and a relative abundance to each genome. The profile and supporting reference sequences form a skeleton definition with which to initialize the weighted random sampling process within a simulation. The user can elect to supply a profile either as an explicit table (Figs 1 and 2) or allow Sim3C to draw abundances at runtime from 1 of 3 distributions (equal abundance, uniformly random, log-normal distribution) for communities made up of strictly mono-chromosomal genomes.

### Error Modelling

Sim3C models 3 forms of experimental noise: machine-based sequencing error, the formation of spurious ligation products, and the contamination of PL libraries with whole genome shotgun (WGS) read-pairs.

To simulate machine-based sequencing error, the paired-end mode from art_illumina [2] has been reimplemented as a Python module (Art.py). This approach was taken as delegating read-pair generation to native invocations of art_illumina proved cumbersome. More explicitly, a loosely coupled solution (via subprocess calls but without an interprocess communication mechanism) lacked sufficient control to generate PL read-pairs in an efficient and robust manner. On the other hand, tightly coupling Sim3C to the ART C/C++ source code (i.e., implementing hooks) would have left Sim3C vulnerable to changes in a non-public external API (i.e., a codebase without formal definition or guarantee of stability). Reimplementation also meant that Art’s many empirically derived machine profiles are available for use by Sim3C, allowing equivalent treatment of machine error when experiments involve both PL (Sim3C) and pure WGS (art_illumina) libraries.

The production of spurious ligation products is an inherent source of noise in PL library construction [19]. Sim3C models spurious pairs as the uniformly random ligation of any 2 cut-sites across all source genomes. While this process disregards cellular organization, it respects the relative abundance of chromosomes. Spurious pairs, and to a lesser extent sequencing error, represent an important confounding signal to downstream analyses that attempt to infer the cellular or chromosomal organization of DNA sequences.

Lastly, conventional WGS read-pairs represent a source of contamination within a PL library that even after Hi-C enrichment steps, are not completely eliminated. The rates at which spurious and WGS read-pairs are injected into a simulation run are controllable by the end-user.

### Simulation modes

Since Hi-C was first introduced [10], the development of variants and extensions has been continual [17,20–22]. Variants have often strived to further enhance the discriminatory power of the original experiment, while seemingly adding yet more complexity to an already challenging protocol (*in situ* DNase Hi-C, sciHi-C) [22]. Others instead have sought compromise, with the aim of lessening the burden on the laboratory (Meta3C). While not considering more recent and complex extensions, Sim3C offers 3 simulation modes: traditional Hi-C, Meta3C, and DNase Hi-C. The first 2 of these modes were chosen as representing the fundamental basis (traditional Hi-C) and an attractive and pragmatic simplification of the original (Meta3C). The third mode (DNase Hi-C) replaces the restriction endonuclease-driven production of the free ends, used to form PL products, with an ideally free process of DNA fragmentation. In the laboratory, this ideally free process could be carried out by DNase digestion or mechanical shearing via sonication.

The most notable difference between the methods of Hi-C and the more recent Meta3C is that after restriction digest, Hi-C employs additional steps leading to the incorporation of biotin tags at each PL junction. This biotinylation permits Hi-C libraries to be subsequently enriched for fragments containing PL junctions by streptavidin-mediated affinity purification. Without enrichment, the simpler Meta3C protocol results in a gross mixture of both WGS and PL read-pairs, where only a small percentage of the total read-pair yield (approx. 1%) will possess PL junctions [23]. The enrichment process within Hi-C, however, is not perfectly efficient, and WGS read-pairs are still observed (approx. 10-50% of reads contain a PL product) [23]. DNase Hi-C replaces restriction digest with a non-specific endonuclease (e.g., DNase I) [24] or mechanical DNA shearing process (e.g., sonication) [20]. In this operational mode, Sim3C treats DNA cleavage as a completely unbiased (free) process, and as such all genomic positions have equal probability of participating in proximity ligation events.

**Figure 3:**
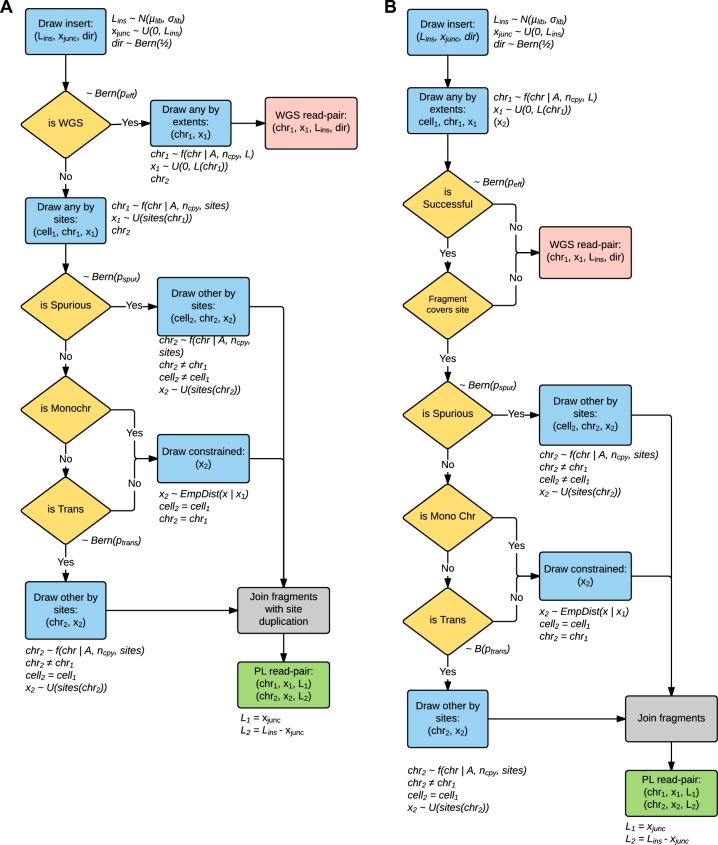
Logical schema used within Sim3C. (**a**) Hi-C and (**b**) Meta3C simulation strategies. Gold diamonds represent simple Bernoulli trials. Blue boxes represent sampling distributions defined by runtime input data (community profile, genomic sequences, enzyme) and the empirically derived distribution for intra-chromosome (*cis*) interaction probability (equation 1). Logical end-points to a single iteration of either algorithm are represented as red (producing a WGS read-pair) and green boxes (producing a PL read-pair). Due to the elimination of the biotinylation step, Meta3C does not produce a duplication of the restriction cut-site overhang (grey boxes).

Within Sim3C, each of the 3 methodological variations is conceptualized as a sequencing strategy (figure 3), and each iteration of a strategy produces 1 read-pair (PL or WGS in origin). For all strategies, an iteration begins by drawing a 3-tuple of insert parameters: length, direction, and junction point (*L_ins_*, *dir, x_junc_*).

After obtaining insert parameters, the Hi-C strategy (Fig. 3a) first tests if the insert will represent a WGS or PL read-pair (∼*Bern*(*p_eff_*)), where efficiency *p_eff_* is defined in the sense of enrichment. When *p_eff_* = 1, there is perfect filtering and all WGS read-pairs are eliminated from the experiment. In the case of WGS, the iteration reaches an end-point and the simulation emits a conventional read-pair drawn from the community definition. In the case of PL, a cut-site 3-tuple is drawn (*gen*_1_, *chr*_1_, *x*_1_), where the categorical distribution over chromosomes is weighted by relative abundances (*A*) and chromosomal copy-numbers (*n_cpy_*), genomic position is sampled uniformly from the set of restriction sites (*sites*(*chr*_1_)), and the parent genome (*gen*_1_) is implicit from the chromosome. Next, a spurious ligation test is performed (∼*Bern*(*p_spur_*)). If a spurious event has occurred, the 3-tuple defining the second cut-site (*gen*_2_, *chr*_2_, *x*_2_) is drawn i.i.d. as the first. If not spurious, next a test for inter-chromosomal (*trans*) ligation is performed. Only source chromosome and position (*chr*_2_, *x*_2_) need be drawn as the second genome is implicitly the same as the first (*gen*_2_ = *gen*_1_). Here, *chr*_2_ is selected without replacement from the set of chromosomes of genome (*gen*_1_) where the categorical distribution is adjusted by removal of *chr*_1_. Finally, an intra-chromosomal (*cis*) ligation must have occurred. As now both genome and chromosome are implicit (*gen*_2_ = *gen*_1_, *chr*_2_ = *chr*_1_), all that is left is to draw genomic position *x*_2_. The pair of positions (*x*_1_, *x*_2_) is constrained by their separation (*s* = |*x*_2_ − *x*_1_|), which is represented by a mixture model of the geometric and uniform distributions (equation 1). This relation possesses rapid falloff with increasing separation and non-zero probability for all chromosomal positions, as has been commonly observed in real experimental data [10,25].

(1) \begin{equation*} Pr(X=s|\alpha , \beta , l) = \beta (1-\alpha )^s \alpha + (1-\beta )/l, \end{equation*}

where β is a mixing parameter, α the geometric distribution shape parameter, and *l* chromosome length.

For Meta3C (Fig. 3 b) after insert parameters are determined, in the same fashion as a regular WGS read, an initial free genomic position is drawn $(chr_1, x^*_1)$, uniformly distributed over the extent of *chr*_1_ rather than only over its cut-sites. In real datasets, it has been observed that neither the restriction digestion nor the re-ligation of free ends is perfectly efficient. Taken as independent probabilities, in our model we conceptualize their joint occurrence as an efficiency factor, *p_eff_*, and a Bernoulli trial (*Bern*(*p_eff_*)) determines whether a sequence read is successful in containing an observable proximity ligation event. Failing this coverage test relegates the iteration and end-point and emits a WGS read-pair. Successful candidates instead continue akin to the Hi-C decision tree, beginning with the test for spurious ligation.

**Figure 4:**
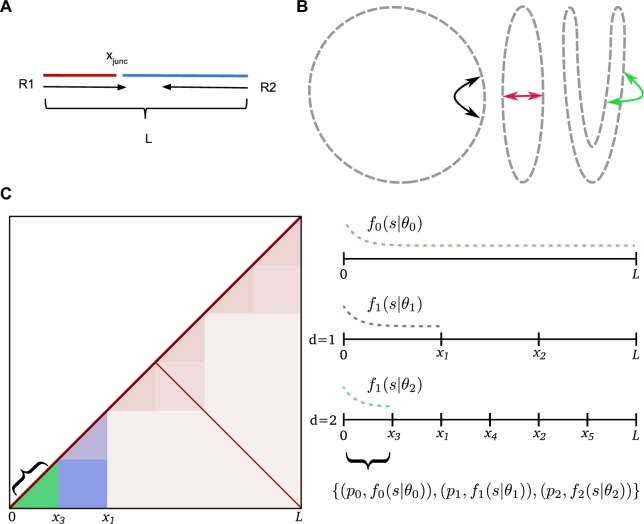
Model details. Generation of proximity ligation inserts (**a**) involves joining 2 randomly drawn parts (red and blue), from which the read-pair (R1, R2) is then simulated. The junction point (*x_junc_*) varies over the interval [0..*L*), and reproduction of read-through events is possible. For an unbounded chromosome (**b**) (circular here), besides strictly primary separation (black arrow), spatial proximity can be induced from successive folding (red, green arrows). When the spatial arrangement is consistent across the population of cells, this will be observable as modulations in the contact frequencies. Sim3C models simple structurally related modulation of observed contact frequencies (**c**). Beyond primary interactions forming the main diagonal, users can reproduce inter-arm-mediated anti-diagonals. Finer-scale modulations attributed to topologically associated domains can optionally be randomly simulated. Primary interactions *f*_0_(*s*|θ_0_) (equation 1) cover the full interval [0, *L*). Each level of recursion (*d* = 1, 2…*n*) generates a finer set of intervals, to which a distribution *f_i_*(*s*|θ_*i*_) and probability *p_i_* are assigned. The final covering of intervals each define a range (green, curly braces) over which a set of probabilities and empirical distribution pairs govern interaction separation *s*.

For both Hi-C and Meta3C, PL read-pairs are produced by joining the free ends drawn above as defined by the fragment parameters (Fig. 4a). Here the location of the PL junction within the insert is determined by *x_junc_*. At the junction, Hi-C differs from Meta3C as the process of biotinylation results in the duplication of the restriction cut-site overhang sequence. The overhang duplication in Hi-C is included in the simulation.

DNase Hi-C is handled similarly to traditional Hi-C, with the exception that, as *in silico* digestion trivially leads to all sites, the simulated digestion is unnecessary to perform and positions can be drawn directly from the uniform distribution over the interval [0..*L_chr_*). Site duplication, attributable to the likely production of random overhangs in this scenario, is not presently simulated.

### Structurally related interactions

Independent of any 3D structure that might exist, the primary and most frequently observed interactions are those that occur along a chromosome (intra-arm) (Fig. 4b), seen as the primary (*y* ≃ *x*) diagonal in the contact map. Sim3C can approximate the less frequent interactions occurring between chromosomal arms (inter-arm) [26], which are visible as anti-diagonal (*y* ≃ *L* − *x*) in the contact map.

At progressively smaller scales, the hierarchical 3D folding of DNA into topologically associated domains (TADs) produces overlapping regions of interaction visible in the contact map as block-like intensity modulations. Though the agents responsible for their formation vary [27,28], the characteristic patterns evident in real data–derived 3C contact maps have been observed across all 3 domains [25,26,29]. Sim3C can optionally approximate the sense of TAD-related modulation by means of a recursive stochastic process.

Our approximation of hierarchical folding begins from the full extent *L* of a chromosome (Fig. 4c). Folding is portrayed by the division of the interval [0..*L*) into a set of non-overlapping sub-intervals {[0, *x*_1_), [*x*_1_, *x*_2_), …, [*x*_*n* − 1_, *x_n_*)}, the number and widths of which are drawn at random (*U*(*l_min_*, *l_max_*), *U*(*n_min_*, *n_max_*)). The procedure is then recursively applied to each sub-interval until a depth *d*, producing a nested set of coverings of the full interval [0..*L*) at progressively finer scales. Across this hierarchical collection, each interval is assigned a uniformly distributed random probability *p_i_* and empirical distribution *f_i_*(*s*|θ_*i*_) (equation 1) for separation *s*, parameterized by shape parameter α_*TAD*_ and interval length *l_inv_* = *x*_*i* + 1_ − *x_i_*, where θ = (α_*TAD*_, β, *l_inv_*).

The process of drawing samples of separation begins by determining the set of intervals {*l_inv_*} that contain an initial point *x*_0_. The intervals, as tuples (*p_i_*, *f_i_*(*s*|θ_*i*_)), then form a categorical distribution (equation 7), from which a governing distribution *f_i_*(*s*|θ_*i*_) is drawn, and finally a sample of separation is taken, *s* ∼ *f_i_*(*s*|θ_*i*_). To efficiently sample from the full collection, an interval-tree data structure is employed. When queried, an interval-tree returns the set of intervals {*l*} overlapping a position *x* in order *O*(log *n* + *m*), where *n* is number of intervals and *m* is number of intervals returned by the query.

(2) \begin{equation*} \mathbf {f} = \left\lbrace f_0(s|\theta _0), f_1(s|\theta _1),\cdots ,f_i(s|\theta _i) \right\rbrace \end{equation*}

(3) \begin{equation*} N = \text{number of distributions} = \left| \mathbf {f} \right| \end{equation*}

(4) \begin{equation*} \mathbf {p} = \left\lbrace p_0, p_1,\cdots ,p_i \right\rbrace \end{equation*}

(5) \begin{equation*} p_i \sim U(0,1) \text{ and } \sum p_i = 1 \end{equation*}

(6) \begin{equation*} n \sim Cat(N, \mathbf {p}) \end{equation*}

(7) \begin{equation*} f(s|n) = \prod _{i=0}^{N-1} f_i(s|\theta _i)^{[i=n]}, \end{equation*}

where [*i* = *n*] is the Iverson bracket.

### Example scenarios

In the following, 3 use cases are presented to demonstrate aspects of the resulting simulation output: bacterial genome, multi-chromosomal eukaryotic (yeast) genome, and metagenome. For each use case, 3C contact maps have been used to pit simulation output against the corresponding real experimental data (table 1).

**Table 1:** Real Hi-C and Meta3C data-sets used within this work

Authors	Type	Method	Accession	Sequencing	Mapped
				details	reads
Beitel et al. [15]	Synthetic bacterial metagenome	Hi-C	SRX377733	MiSeq 160bp PE insert range: 280-420bp enzyme: HindIII	20552775
Burton et al. [16]	Synthetic yeast metagenome	Hi-C	SRX527868	HiSeq2500 100bp PE insert range: 450-550bp enzyme: HindIII	9704944
Le et al. [26]	Single bacterial genome	Hi-C	SRX263925	HiSeq2000 40bp PE insert range: 200-600bp enzyme: NcoI	22324360
Marbouty et al. [41]	Synthetic bacterial metagenome	Meta3C	doi:10.5061/ dryad.gv595	HiSeq2000 100bp PE insert range: 400-800bp enzyme: HpaII	7975740

The total off-diagonal weight of the contact map was used to calibrate the amount of simulated sequencing required to approximately match the outcome of the real experiments.

### Bacterial

A monochromosomal bacterial genome is perhaps the simplest scenario to which proximity ligation methods have been applied, making for a sensible entry point from which to make comparison. Due to the smaller extent, a bright and high-resolution contact map (10-kbp bin size) is possible for a practical volume of sequencing data, potentially revealing fine detail not easily discerned with larger bin sizes (50–100-kbp bin size).

The genome of *Caulobacter crescentus* NA1000, a model organism in the study of cellular differentiation and regulation of the cell cycle, is comprised of a single 4-Mbp circular chromosome [30]. Deep Hi-C sequencing of *C. crescentus* has been used to explore the degree to which bacterial chromosomes can be regarded as organized and provided evidence for the existence of so-called chromosomal interaction domains (CIDs) [26]. As a prokaryotic analog of topologically associated domains from eukaryotic literature [28,31–32], these regions are believed to promote intra-domain loci interactions and thereby act to functionally compartmentalize the genome. This chromosomal structure was observed to be at once disruptable through rifampicin-mediated inhibition of transcription and malleable by the movement of highly expressed genes [26].

**Figure 5:**
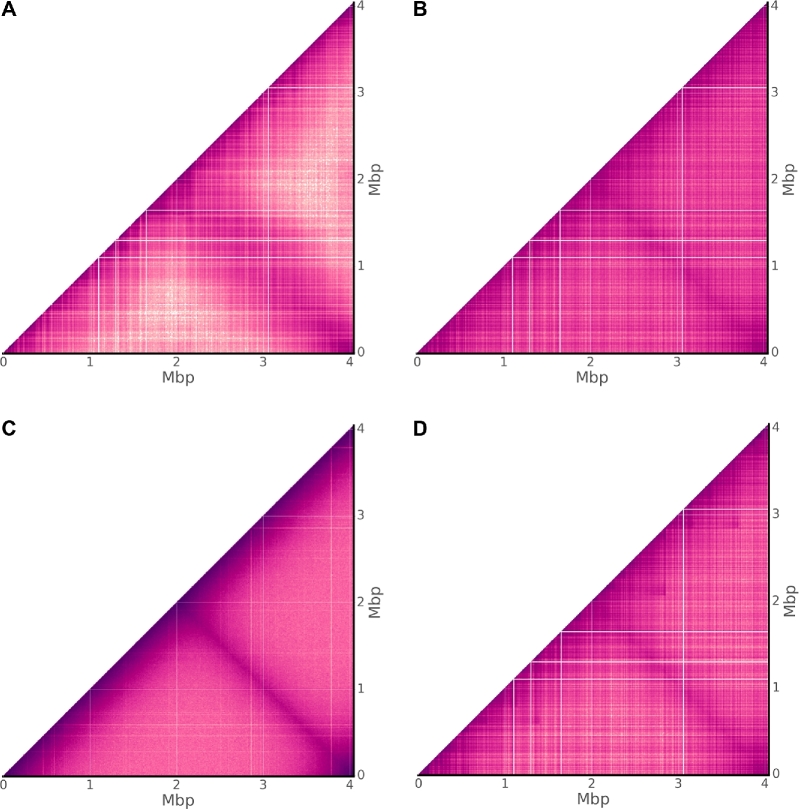
Bacterial contact maps. Observed Hi-C interactions for the monochromosomal genome of *Caulobacter crescentus* NA1000. Comparing (**a**) real experimental data [26] with the 3 simulation choices (**b**) traditional Hi-C, (**c**) DNase Hi-C, and (**d**) traditional Hi-C with TADs enabled. Sharp rectilinear modulations of the intensity within (a) and (b) indicate a reduction in PL observations within a given bin. Not due to 3D chromosome structure, rather such features can be attributed largely to mappability and low cut-site density. (c) Without an enzymatic constraint, a significantly smoother field is apparent, yet still susceptible to mappability. (d) Enabling topologically associated domains highlights the similarity between features produced merely from biases and what could be truly associated with 3D structure.

For the raw contact map of *C. crescentus*, prominent rectilinear features are apparent for both real and simulated traditional Hi-C sequencing data (Fig. 5a,b), while notably for simulated unrestricted Hi-C the field is much smoother (Fig. 5c). Within the Sim3C model, a single distribution governs both intra- and inter-arm interactions. Inspection of the real-data contact map (Fig. 5a) suggests that the true relationship governing inter-arm interactions is more dispersed. This perhaps is not surprising, where different arms associating spatially possess a greater number of potential configurations than can be taken on by the primary chromosome backbone. Additionally, for the real contact map, long-range interactions away from either diagonal can be seen to drop to a lower threshold than that produced from simulation.

Within the unrestricted Hi-C map, the fine zero-intensity rectilinear features are a direct result of poor mappability (non-unique sequence), where their small size reflects the extent of the non-unique regions (example: rRNA genes) and the single base-pair resolution of the less constrained read generation process. The process of enzymatic digestion is the only difference between the unrestricted and traditional Hi-C simulation models. The clear contrast in their contact maps is thus a combination of factors either directly inherent to digestion (cut-site density) or a byproduct of downstream bioinformatics analysis (e.g., filtering heuristics). Though the problem of mappability exists for any reference-based representation, for real and simulated traditional Hi-C, zero-intensity rectilinear features mark regions devoid of cut-sites over at least 10 kbp.

Enabling TAD approximation in simulated traditional Hi-C (Fig. 5d) has the effect of modulating map intensity in a manner not particularly distinct from that produced purely from experimental/workflow bias. Discriminating between these 2 feature sources—one representing experimental signal, the other representing noise—demands attention when developing solutions to problems such as normalization. Contact map normalization methods, whether based upon explicit or implicit bias models [33], may leave behind remnants of noise-related features from either a lack of convergence or model limitations. Downstream inferencing should therefore not be made under an assumption of bias-free signal.

### Eukaryotic

The 8 chromosomes of the 15.4-Mbp genome of the native xylose-fermenting yeast *Scheffersomyces stipitis* CBS 6054 [34] range in size from 970 kbp to 3.5 Mbp. The organism was 1 of 16 yeasts included in a synthetic community to explore the application of Hi-C sequencing to deconvolving metagenomic assemblies [16], and it is divergent enough from other synthetic community members to permit unambiguous read mapping, and thus act as a proxy for a clonal experiment.

**Figure 6:**
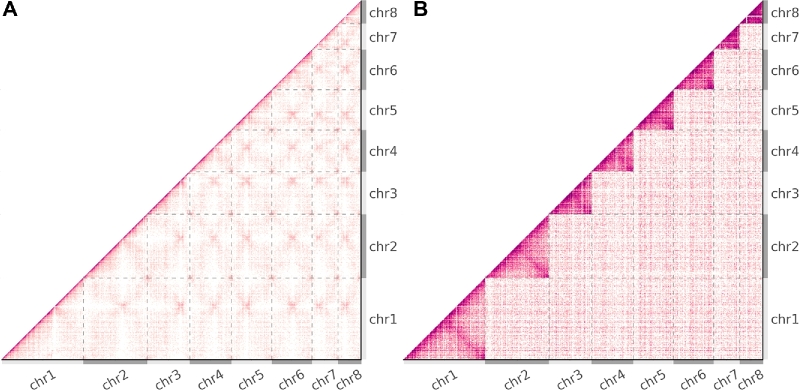
Eukaryotic contact maps. Observed Hi-C interactions of (**a**) real and (**b**) simulated data from the 8 chromosome genomes of the budding yeast *Scheffersomyces stipitis* CBS 6054 [16]. Grey dashed lines and alternating light and dark grey axes demarcate the boundaries between chromosomes. (b) Simulated data elicit a flat field, and the clearly evident higher rate of intra- to inter-interactions makes for easily observable chromosomal boundaries within the map. (a) Contrastingly for real data, the similar rates of intra-chr and inter-chr interactions reveal the physical constraints imposed by centromere-SPB tethering on all 8 chromosomes [35].

From the contact map of real Hi-C data (Fig. 6a), it can be seen that the rates of intra-chromosomal and inter-chromosomal interactions are roughly equivalent in magnitude. Across the 8 chromosomes of *S. stipitis*, there is significant uniformity in the degree of physical intimacy within and between all chromosomes. The subtleties of this chromosomal organization reveal a self-similar “fuzzy-x” pattern repeated between all chromosomes across the contact map. The convergence point within the pattern is attributed to centromere-SPB binding and has been used to predict centromere locations [35]. It has been shown that the physical constraints generated from the interaction of centromeres to the spindle pole body (SPB) and telomeres to the nuclear envelope are sufficient to explain a number of experimental observations in real data [36,37]. As Sim3C was derived from the study of bacterial datasets, our simulation model does not currently include a notion of these higher organism physical constraints. Consequently, the contact map derived from simulated traditional Hi-C sequencing elicits a flat field (Fig. 6b), where the intensity variation that does exist is a byproduct of aforementioned factors such as mappability and cut-site density. For the runtime parameters employed, the rate of intra-chromosomal contact is higher than that of inter-chromosomal, making clear the boundaries between the 8 chromosomes (Fig. 6b). Though our model is presently incomplete for higher organisms, there remains a potential utility as an analytical or simply observational prior.

### Metagenomic

**Figure 7:**
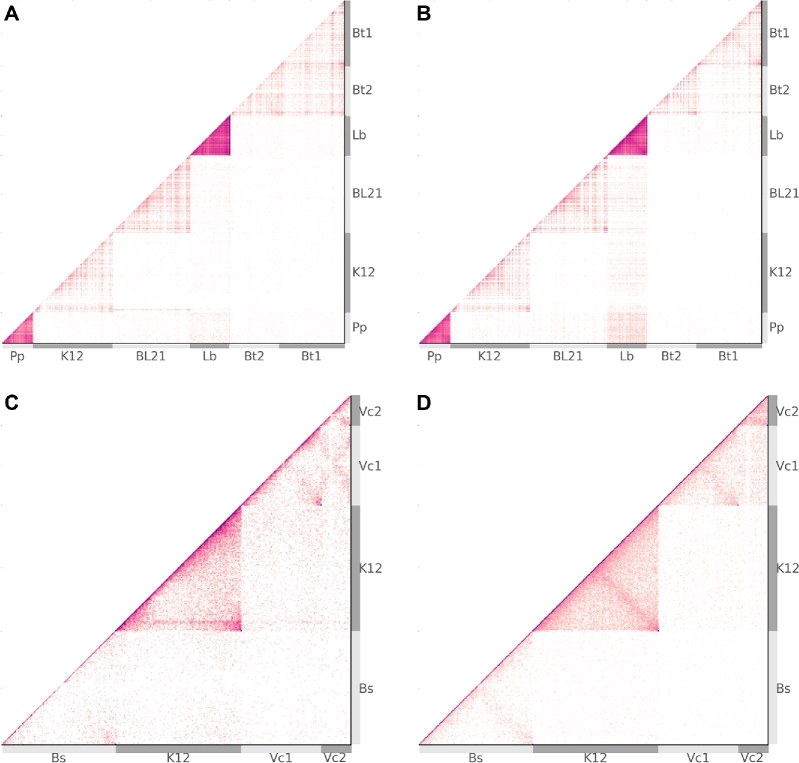
Metagenomic contact maps. From synthetic microbial communities, raw contact maps from real (**a**) and simulated (**b**) traditional HiC, and real (**c**) and simulated (**d**) Meta3C. Chromosome boundaries are demarcated by alternating light and dark grey bands (Tables 2, 3), while the small plasmids of *L. brevis* are omitted for clarity. Although the original works [15,17] intended uniform abundance, the results exhibit significant variation in abundance. Lysis efficiency (not modelled) and enzyme suitability are significant factors contributing to the overall intensity of a given chromosome. For more abundant members of the Hi-C community (*P. pentosaceus* and *L. brevis*), signal due only to spurious ligation can appear to suggest inter-cellular interactions when none are present (b).

In the deconvolution of metagenomes, proximity ligation methods hold great potential as new sources of information and have been investigated by the construction and sequencing of synthetic communities [15–17]. We selected 2 previously constructed synthetic bacterial communities, 1 employing traditional Hi-C and the other Meta3C (table 1). Intended as “proof of concept” experiments, neither community reflects a real environment, but rather they were intended to be easily interpreted and include interesting features, such as range of GC, single and multi- chromosomal genomes, and strain-level divergence. The Hi-C community involved 5 genotypes from 4 species, 1 genome of 2 chromosomes (*B. thailandensis*), *E. coli* strains BL21 and K12 (average nucleotide identity [ANI], 99%), and a wide overall GC range of 37–68% (Table 2). Of lower complexity, the Meta3C community involved 3 genomes from 3 species, included 1 genome of 2 chromosomes (*V. cholerae*), and had a narrower GC range of 44–51% (table 3). Relative to the single genome experiments above, a lower depth of sequencing resulted in a lower overall contact map intensity (Fig. 7). This is particularly the case for Meta3C, where, by the nature of the method, a large proportion (approx. 99%) of the sequencing yield is in reality conventional WGS read-pair data [17]. As a direct result, in binning the Meta3C dataset, there were insufficient counts to fully establish finer detail within the contact maps, leaving a smoother appearance.

**Table 2:** Synthetic Hi-C community

Name	Replicons	Accession	Chr abbr.	*A*	*n_cpy_*	%GC	*n_x_*	*m*
		NC_007651	Bt1			67.29	225	0.24
*Burkholderia*	2			0.054	1			
		NC_007650	Bt2			68.07	144	0.20
*thailandensis* E264								
*Escherichia coli* BL21	1	NC_012892	BL21	0.242	1	50.83	508	0.46
*Escherichia coli* K12	1	NC_010473	K12	0.166	1	50.78	568	0.50
DH10B								
		NC_008497	Lb			46.22	629	1.12
*Lactobacillus brevis*	3	NC_008498	-	0.436	1	38.64	3	0.92
ATCC 367		NC_008499	-			38.51	16	1.84
*Pediococcus pentosaceus*	1	NC_008525	Pp	0.102	1	37.36	863	1.93
ATCC 25745								

A synthetic community used to demonstrate the utility of Hi-C sequencing data in resolving a microbial metagenome [15]. It is composed of 5 bacteria, including 2 closely related strains (*E. coli* K12 and BL21), a genome with 2 plasmids (*L. brevis*), and a 2-chromosome genome (*B. thailandensis*). A is relative abundance, *n_cpy_* is copy number, *n_x_* is number of restriction sites, and *m* = *n_x_*/*n*_0_ is match quality between chromosome and enzyme choice: *m* < 1 is worse, *m* > 1 is better.

**Table 3:** Synthetic Meta3C community

Name	Replicons	Accession	Chr abbr.	*A*	*n_cpy_*	%GC	*n_x_*	*m*
*Bacillus subtilis* subsp.	1	NC_000964	Bs	0.123	1	43.51	14529	0.88
subtilis str. 168								
*Escherichia coli* str.	1	NC_000913	K12	0.562	1	50.79	24311	1.34
K-12 substr. MG1655								
		NC_002505	Vc1			47.70	5909	0.51
*Vibrio cholerae* O1	2			0.332	1			
		NC_002506	Vc2			46.91	1802	0.43
biovar El Tor str.								
N16961								

A synthetic community used to demonstrate the utility of Meta3C sequencing data in resolving a microbial metagenome [17,41]. It is composed of 3 bacteria with 1 possessing 2 chromosomes. *A* is relative abundance, *n_cpy_* is copy number, *n_x_* is number of restriction sites, and *m* = *n_x_*/*n*_0_ is match quality between chromosome and enzyme choice: *m* < 1 is worse, *m* > 1 is better.

As with single-genome experiments, metagenomic contact maps are locally modulated by factors such as mappability and cut-site density. Importantly now for metagenomes, the factors of relative abundance and GC content interact to alter the observed intensity of each chromosome within the contact map.

As a first approximation and assuming agreement in nucleotide sampling frequency, we expect *n*_0_ = *L*/4^λ^ recognition sites for an enzyme of site length λ and DNA sequence length *L*. The degree to which an enzyme and DNA sequence deviate from this estimate could be described as how well they match, *m* = *n_x_*/*n*_0_. Poorer quality matches (*m* < 1) occur when an enzyme’s recognition site is underrepresented, while conversely, better quality matches (*m* > 1) describe a situation of more recognition sites than expected.

When multiple chromosomes are taken as a community, the relative proportion of sites from each represents an observational bias when conducting 3C-based experiments. For community *C*, the number of sites *n_x_* from chromosome *x* determines the number of potential PL pairings *N_x_* within *C* that *x* (equation 8). The number of intra-chromosomal and inter-chromosomal potential pairs thus respectively vary quadratically and linearly with *n_x_*. Regarding the process of observing a PL event (read-pair) from the community as a random draw with replacement, and the selection pool as comprised of all potential events from all chromosomes, variation in match quality constitutes a per-chromosome bias. In real laboratory experiments, the composition of the selection pool is further modified by variation in other factors, such as cellular lysis efficiency, unintended DNA fragmentation, and relative abundance. In particular, when relative abundances A are introduced, the odds of observing a PL event involving chromosome *x* are then proportional to the product *p_x_* ∝ *A_x_N_x_*/*N_C_*. Although the processes of intra-chromosomal, inter-chromosomal, and inter-cellular (spurious) ligation are treated independently in our simulation model, in this manner, per-chromosome intensity (observation rate of chromosome *x*) can vary significantly within a metagenome.

(8) \begin{equation*} N_x=n_x^2 + n_x \sum _{n_y \in C \setminus n_x} n_y \end{equation*}

Though the original laboratory experiments reported by Beitel et al. [15] and Marbouty et al. [17] intended to create synthetic communities with uniform relative abundances, in practice each possesses a non-uniform profile. The variation in GC content is largest for the Hi-C experiment and, together with non-uniform relative abundances, produces a wide range of chromosome intensity for both real and simulated data (Fig. 7a,b). For both the real and simulated Hi-C maps, the frequent observation of PL events involving *P. pentosaceus* (Pp) and *L. brevis* (Lb) suggests the possibility that inter-cellular interaction is significant. Within the simulated map at least, inter-cellular pairs are produced exclusively through the process of spurious ligation (noise) and are observed at a higher rate than in the real data, indicating that, as expected, spurious ligation rates across species are correlated with their relative abundances.

Further for the Hi-C data, the 2-chromosome genome of *B. thailandensis* (Bt1, Bt2) (Fig. 7a) has a greater rate of inter-chromosomal interaction than expected from comparing it with simulation (Fig. 7b). Meanwhile, the clear delineation of *E. coli* strains BL21 and K12 (*ANI* > 99%), with little inter-cellular signal, helps to support the notion that the inter-chromosomal interactions observed between *B. thailandensis* chromosomes (*ANI* ≃ 83%) are real and not a by-product of inadequate filtering.

### Limitations and future work

Sim3C in its current form has several limitations, some of which present opportunities for future work. Sim3C’s repertoire of structural features is currently limited to those found in microbes—circular and linear chromosomes with randomly generated approximations of self-associating domains (CIDs/TADs). Sim3C does not model structural features observed in larger, more complex genomes (CTCF/cohesin loops, A/B compartments, chromosome territories) [10,38]. Such features are becoming increasingly well characterized [39], and a simulator capable of modelling these features would surely be valuable. Mammalian genomes are much larger than microbial genomes, however, and additional work to improve the scalability of Sim3C will likely be required.

Some features of microbial eukaryotes, such as the point centromeres found in budding yeast genomes [40], are computationally simpler [35,36] yet remain unmodelled in Sim3C. The addition of these sorts of model details would be best supported by introducing model initialization via external data (experimental observations, motif detection, cell phase), which subsequently would require extension of the community profile definition. Careful design would be required to ensure these features could be added without compromising ease of use.

## Methods

### Reference Data

To compare Sim3C against real experiments, we obtained previously published experimental read-pair datasets (Table 1) and their accompanying reference genomes (Tables 2, 3) from public archives. In the case of the single genome project of *Caulobacter crescentus* CB15 [26], sequencing data derived from untreated swarmer cells was chosen and the laboratory strain *C. crescentus* NA1000 (acc: NC_011916) was used as the reference genome. For the yeast genome, the completed 8-chromosome genome of *Scheffersomyces stipitis* CBS 6054 was used as a reference (acc: PRJNA18881), and the respective reads were extracted from the MY16 yeast synthetic metagenome [16] by direct mapping with BWA MEM. Extraction by mapping in isolation was employed as *S. stipitis* was the second furthest phylogenetically removed yeast in the synthetic community and was the most contiguous (N50: 60 kbp) from the whole synthetic community *de novo* metagenomic WGS assembly.

### Read generation

Experimental parameters used in read simulation were set to agree as closely as reasonably possible to the respective real experiments, employing the same read length and restriction enzyme (Table 1). In each experiment, the published fragment size range was approximated by a normal distribution (Table 4). For ease of reproducibility, a single random seed (1234) was used in all simulations. As our intent was primarily to demonstrate functionality, rates of inter-chromosomal and spurious events were adjusted per-experiment only through a qualitative process. For simulation of metagenomic datasets, relative abundances were estimated by mapping real experimental reads to the respective reference genomes. From each real experiment, the off-diagonal weight of the resulting contact map was used to calibrate the amount of simulated sequencing required to achieve roughly equivalent intensity (Table 4). Both real and simulated read-pair datasets were mapped to their respective reference genomes using BWA MEM (v0.7.15-r1140, RRID:SCR_010910) [42].

**Table 4:** Runtime simulation

Experiment	Insert μ (bp)	Insert σ (bp)	Anti rate	Spurious rate	Trans rate	Reads (×10^6^)
Beitel et al.	300	50	0.2	0.05	0.1	7
Burton et al.	400	50	0.2	0.5	0.15	1.5
Le et al.	400	100	0.2	0.2	0.1	22
Marbouty et al.	600	100	0.2	0.2	0.2	7.5

Parameters supplied to Sim3C during read generation.

### Contact maps

Contact maps were produced using our own tool (contact_map.py), where heatmap intensity was plotted as log-scaled observational frequency. All aligned reads were subject to the same basic filtering criteria: BWA MEM mapq >5 and alignment length ≥50% of read length, with the added restriction that read alignments must have begun with a match. For methods that employed a restriction enzyme (traditional Hi-C, Meta3C), we constrained the maximum allowable distance from an aligned read to the nearest upstream cut-site. Calculated per chromosome, this distance constraint could not exceed 2-fold the median cut-site spacing. Rather than simply delete the primary diagonal for the sake of reducing the displayed dynamic range in figures, we instead reduced its intensity by categorizing properly paired reads with an estimated fragment size of less than 2 of the reported mean as being conventional WGS (non-PL) reads and ignored them. The resolution of contact maps was adjusted between experiments so as to present a sufficiently bright image without undue loss of resolution. The contact map bin sizes employed were: 10 000 bp for the single bacterial genome, 25 000 bp for the yeast genome, and 40 000 bp for the Hi-C and Meta3C metagenomes (Tables 2, 3).

## Availability of data and materials

Snapshots of the supporting code are available from the *GigaScience* repository, *Giga*DB [43].

## Availability of supporting source code and requirements

- Project name: Sim3C
- Release version: 0.1
- Project homepage: https://github.com/cerebis/sim3C
- RRID: SCR_015772
- DOI: 10.5281/zenodo.1030812
- Operating system: platform independent
- Programming languages: Python 2.7
- License: GNU GPL v3

## Abbreviations

PL: proximity ligation;
WGS: whole genome shotgun
CID: chromosomal interaction domain
TAD: topologically associated domain
*Bern*(*x*): Bernoulli distribution
*U*(*x*): uniform distribution
*N*(μ, σ): normal distribution
*cis*: intra-chromosomal
*trans*: inter-chromosomal

## Competing interests

The authors declare that they have no competing interests.

## Funding

This work was supported under the Australian Research Council’s Discovery Projects funding scheme (project number: LP150100912, CI: S.P. Djordjevic). The NeCTAR Research Cloud is an Australian Government project conducted as part of the Super Science Initiative and financed by the Education Investment Fund (EIF) and National Collaborative Research Infrastructure Strategy (NCRIS).

- https://www.education.gov.au/education-investment-fund
- https://www.education.gov.au/national-collaborative-research-infrastructure-strategy-ncris

## Authors contributions

M.D. designed and implemented Sim3C and wrote the manuscript and prepared figures. A.D. assisted in the design and contributed to the manuscript.

## Supplementary Material

GIGA-D-17-00126_Original-Submission.pdf

GIGA-D-17-00126_Revision-1.pdf

GIGA-D-17-00126_Revision-2.pdf

Response-to-Reviewer-Comments_Original-Submission.pdf

Response-to-Reviewer-Comments_Revision-1.pdf

Reviewer-1-Report-(Original-Submission).pdf

Reviewer-1-Report-(Revision-1).pdf

Reviewer-2-Report-(Original-Submission).pdf

Reviewer-2-Report-(Revision-1).pdf
