# Supplementary material for: Sim3C: simulation of Hi-C and Meta3C proximity ligation sequencing technologies
Source: Gigascience. 2017 Nov 15;7(2):1–12. doi: 10.1093/gigascience/gix103 (PMC5827349; doi:10.1093/gigascience/gix103)
Supplement: GIGA-D-17-00126_Revision-2.pdf [file gix103_giga-d-17-00126_revision-2.pdf]

GigaScience

Sim3C: Simulation Of HiC And Meta3C Proximity Ligation Sequencing Technologies

--Manuscript Draft--

**Manuscript Number:**	GIGA-D-17-00126R2
**Full Title:**	Sim3C: Simulation Of HiC And Meta3C Proximity Ligation Sequencing Technologies
**Article Type:**	Technical Note
**Funding Information:**	Australian Research Council (LP150100912) — Dr Aaron E. Darling
**Abstract:**	Background<br><br>Chromosome conformation capture (3C) and Hi-C DNA sequencing methods have rapidly advanced our understanding of the spatial organization of genomes and metagenomes. Many variants of these protocols have been developed, each with their own strengths. Currently there is no systematic means for simulating sequence data from this family of sequencing protocols, potentially hindering the advancement of algorithms to exploit this new datatype.<br><br>Findings<br><br>We describe a computational simulator that, given simple parameters and reference genome sequences, will simulate Hi-C sequencing on those sequences. The simulator models the basic spatial structure in genomes that is commonly observed in Hi-C and 3C datasets, including the distance-decay relationship in proximity ligation, differences in the frequency of interaction within and across chromosomes, and the structure imposed by cells. A means to model the 3D structure of randomly generated topologically associating domains (TADs) is provided. The simulator considers several sources of error common to 3C and Hi-C library preparation and sequencing methods, including spurious proximity ligation events and sequencing error.<br><br>Conclusions<br><br>We have introduced the first comprehensive simulator for 3C and Hi-C sequencing protocols. We expect the simulator to have use in testing of Hi-C data analysis algorithms, as well as more general value for experimental design, where questions such as the required depth of sequencing, enzyme choice, and other decisions can be made in advance in order to ensure adequate statistical power with respect to experimental hypothesis testing.
**Corresponding Author:**	Aaron Darling<br><br>AUSTRALIA
**Corresponding Author Secondary Information:**	
**Corresponding Author's Institution:**	
**Corresponding Author's Secondary Institution:**	
**First Author:**	Matthew Z. DeMaere
**First Author Secondary Information:**	
**Order of Authors:**	Matthew Z. DeMaere<br>Aaron E. Darling
**Order of Authors Secondary Information:**	
**Response to Reviewers:**	We have made the requested minor revisions.
**Additional Information:**	

Powered by Editorial Manager® and ProduXion Manager® from Aries Systems Corporation

Question	Response
Are you submitting this manuscript to a special series or article collection?	No
**Experimental design and statistics**<br><br>Full details of the experimental design and statistical methods used should be given in the Methods section, as detailed in our Minimum Standards Reporting Checklist. Information essential to interpreting the data presented should be made available in the figure legends.<br><br>Have you included all the information requested in your manuscript?	Yes
**Resources**<br><br>A description of all resources used, including antibodies, cell lines, animals and software tools, with enough information to allow them to be uniquely identified, should be included in the Methods section. Authors are strongly encouraged to cite Research Resource Identifiers (RRIDs) for antibodies, model organisms and tools, where possible.<br><br>Have you included the information requested as detailed in our Minimum Standards Reporting Checklist?	Yes
**Availability of data and materials**<br><br>All datasets and code on which the conclusions of the paper rely must be either included in your submission or deposited in publicly available repositories (where available and ethically appropriate), referencing such data using a unique identifier in the references and in the "Availability of Data and Materials" section of your manuscript.<br><br>Have you have met the above requirement as detailed in our Minimum Standards Reporting Checklist?	Yes

Sim3C: simulation of Hi-C and Meta3C proximity ligation sequencing technologies

Matthew Z DeMaere[1], Aaron E Darling[1*],

1 The ithree institute, University of Technology Sydney (Sydney, NSW, Australia)

*** aaron.darling@uts.edu.au**

Abstract

Background

Chromosome conformation capture (3C) and Hi-C DNA sequencing methods have rapidly advanced our understanding of the spatial organization of genomes and metagenomes. Many variants of these protocols have been developed, each with their own strengths. Currently there is no systematic means for simulating sequence data from this family of sequencing protocols, potentially hindering the advancement of algorithms to exploit this new datatype.

Findings

We describe a computational simulator that, given simple parameters and reference genome sequences, will simulate Hi-C sequencing on those sequences. The simulator models the basic spatial structure in genomes that is commonly observed in Hi-C and 3C datasets, including the distance-decay relationship in proximity ligation, differences in the frequency of interaction within and across chromosomes, and the structure imposed by cells. A means to model the 3D structure of randomly generated topologically associating domains (TADs) is provided. The simulator considers several sources of error common to 3C and Hi-C library preparation and sequencing methods, including spurious proximity ligation events and sequencing error.

Conclusions

We have introduced the first comprehensive simulator for 3C and Hi-C sequencing protocols. We expect the simulator to have use in testing of Hi-C data analysis algorithms, as well as more general value for experimental design, where questions such as the required depth of sequencing, enzyme choice, and other decisions can be made in advance in order to ensure adequate statistical power with respect to experimental hypothesis testing.

Keywords

Hi-C, Meta3C, 3C, DNA sequencing, simulation, metagenomics

Findings

Software testing

To the casual observer, formal software testing is often thought to begin and end with the validation of fine-grained behavioural (functional) aspects; such as the correct execution of individual methods. In day to day use however, what can matter most to end-users are broader system attributes such as speed, scalability, reproducibility and ease of use. To ensure a project offers maximum value, a thorough testing process would collectively examine all aspects.

For inferential software within scientific fields, the system-level attributes of precision and accuracy are of primary interest, and their quantification is best accomplished by comparison to a known truth (gold standard). Therefore, any testing methodology capable of providing an *a priori* gold standard, particularly without estimation, improves this facet of testing significantly.

Purpose-built bioinformatics software ultimately acts on experimentally collected observations. The inherent noise and variation that comes with experimental data means achieving testing thoroughness is a great challenge. Ready access to sufficient data sources is a fundamental necessity for adequate software testing.

For established experimental methods, public data archives are a first choice for the necessary testing data. When high quality metadata is available, testing driven by real data becomes possible. However, even when sufficient depth and description of data is available, difficulty can remain in matching desired test data characteristics to what actually exists in one or several public dataset(s). Further, fine-grained whole-corpus querying of metadata on remote data archives is not always possible, frequently making the up-front job of data selection a difficult task. Once selected, obtaining said real data can be time-consuming or even infeasible in locations with lower network speeds and/or high bandwidth costs. In advancing fields such as DNA sequencing, new experimental datatypes can appear for which the public data archives contain only a handful of examples and few researchers would have the time and financial resources to commit to experimental generation of new data purely for software testing.

Though performance on real data is the ultimate arbiter of analytical value, advantaged by explicit control over its characteristics, a faithful simulation of real data can act as a valuable proxy. Simulation-driven development and testing has proven to be a highly cost effective and time efficient approach. It offers the possibility to explore a near continuum of data characteristics, subjecting software to an otherwise unavailable degree of testing thoroughness. Certainty and control makes attaining the twin objectives of rigorous testing and an *a priori* gold standard straightforward. This enables us not only to be more certain about when we have failed, but also to extrapolate this process to infer the limits of success within the experimental parameter space.

Tools for simulating DNA sequencing reads have existed from the very early days of genomics, beginning with the many anonymous implementations of simple DNA shearing algorithms, up to the most recent highly detailed empirical model simulators [14, 15, 20, 31]. From read simulation in isolation, field advancements such as metagenomics have been accompanied soon after by simulators reflecting their specific data characteristics and evolving experimental methodology [2, 17, 37].

We introduce Sim3C, a software package designed to simulate data generated by Hi-C and other 3C-based proximity ligation (PL) sequencing protocols. The software includes flexible support for a range of sequencing project scenarios and choice of three 3C methods (Hi-C, Meta3C, DNase Hi-C). The resulting output (paired-end FastQ) is easily assimilated into existing analysis workflows. It is our intention that Sim3C provide the Hi-C/3C research community with means to further validate existing

software projects, to support new experimental or analysis development initiatives and as a platform for exploration, such as the comparative analysis of clustering algorithms [9].

3C sequencing

3C-based sequencing protocols, including Hi-C, 4C-seq, and Meta3C, have great potential to address questions directed at the spatial organization of DNA in samples ranging from eukaryotic tissue, to single cells, to microbial communities. The growing use of these protocols creates a legitimate need for a simulator capable of generating data with relevant characteristics.

Chromosome conformation capture (3C) was originally designed as a PCR-based assay to measure interactions among a small number of defined regions of eukaryotic chromosomes [8]. In 2009 Lieberman-Aiden [22] reported an extension of the protocol to high throughput sequencing, enabling the global spatial arrangement of chromosomes to be reconstructed at unprecedented resolution. All 3C protocols depend on an initial formalin fixation step, which crosslinks proteins bound to DNA in vivo. Subsequently cells are lysed and the DNA:protein complexes are sheared enzymatically and/or physically to create free ends in the bound DNA strands. These free ends are then subjected to a proximity ligation reaction, in which ligation of free ends preferentially occurs among DNA strands cobound in a protein complex. The DNA:protein crosslinks are then reversed, the DNA is purified, and an Illumina-compatible sequencing library is constructed. In Hi-C protocols, the proximity ligation junctions can then be further purified in the sequencing library.

3C-derived methods have found several applications beyond their initial use to reconstruct 3D chromosome structure. For example, it has been shown that 3C-derived data provide a valuable signal for genome scaffolding [5, 11], as well as a signal that can support genome-wide haplotype phasing [18, 39]. 3C-derived data has also proven valuable for metagenomics, where initial studies on mock communities demonstrated that highly accurate genome reconstruction in mixed microbial communities could be facilitated by proximity ligation sequence data [4, 6, 27]. Subsequent application to naturally occurring microbial communities has also suggested that bacteriophage can be linked to their hosts with this data type [25].

In the remainder of this manuscript we describe the Sim3C software and demonstrate how it can be used to simulate data for various 3C-derived experiments.

Experiment scenarios

Beyond simple monochromosomal genome sequencing experiments, Sim3C offers support for the more complex scenarios of multi-chromosomal genomes and metagenomes. A scenario is defined by way of a community profile; assigning a copy-number and containing genome to each chromosome and a relative abundance to each genome. The profile and supporting reference sequences form a skeleton definition with which to initialize the weighted random sampling process within a simulation. The user can elect to supply a profile either as an explicit table (listing 1, 2) or allow Sim3C to draw abundances at runtime from one of three distributions (equal abundance, uniformly random, log-normal distribution) for communities made up of strictly mono-chromosomal genomes.

```
#chrom  cell  abund  copynum
chr1    bac1  0.4    1
plas1   bac1  0.4    1
chr2    bac2  0.6    1
```

Listing 1. A mock two genome community. For demonstration purposes, we assume that the plasmid (plas1) is present in four copies and that there is a 0.4/0.6 relative abundance split between the two organisms (bac1, bac2) in the community

```
#chrom  cell  abund  copynum
chr1    euk1  1      1
chr2    euk1  1      1
chr3    euk1  1      1
chr4    euk1  1      2
```

Listing 2. A mock four chromosome genome. Cellular abundance is a constant across the profile, while chr4 exists in two copies. Note that relative abundances specified in a profile are not required to sum to 1, but are normalised internally.

Error Modelling

Sim3C models three forms of experimental noise: machine-based sequencing error, the formation of spurious ligation products and the contamination of PL libraries with WGS read-pairs.

To simulate machine-based sequencing error, the paired-end mode from art_illumina [15] has been reimplemented as a Python module (`Art.py`). This approach was taken as delegating read-pair generation to native invocations of art_illumina proved cumbersome. More explicitly, a loosely coupled solution (via subprocess calls but without an IPC mechanism) lacked sufficient control to generate PL read-pairs in an efficient and robust manner. On the other hand, tightly coupling Sim3C to the ART C/C++ source code (i.e. implementing hooks) would have left Sim3C vulnerable to changes in a non-public external API (i.e. a codebase without formal definition or guarantee of stability). Reimplementation also meant Art's many empirically derived machine profiles are available for use by Sim3C, allowing equivalent treatment of machine-error when experiments involve both PL (Sim3C) and pure WGS (art_illumina) libraries.

The production of spurious ligation products is an inherent source of noise in PL library construction [29]. Sim3C models spurious pairs as the uniformly random ligation of any two cut-sites across all source genomes. While this process disregards cellular organisation, it respects the relative abundance of chromosomes. Spurious pairs, and to a lesser extent sequencing error, represent an important confounding signal to downstream analyses that attempt to infer the cellular or chromosomal organisation of DNA sequences.

Lastly, conventional WGS read-pairs represent a source of contamination within a PL library, which even after Hi-C enrichment steps, are not completely eliminated. The rates at which spurious and WGS read-pairs are injected into a simulation run are controllable by the end-user.

Simulation modes

Since Hi-C was first introduced [22], the development of variants and extensions has been continual [12, 27, 34, 35]. Variants have often strived to further enhance the discriminatory power of the original experiment, while seemingly adding yet more complexity to an already challenging protocol (*in-situ* DNase Hi-C, sciHi-C) [35]. Others instead have sought compromise, with the aim of lessening the burden on the laboratory (Meta3C). While not considering more recent and complex extensions, Sim3C offers three simulation modes: traditional Hi-C, Meta3C and DNase Hi-C. The first two of these modes were chosen as representing the fundamental basis (traditional Hi-C) and an attractive and pragmatic simplification of the original (Meta3C). The third mode (DNase Hi-C) replaces the restriction endonuclease driven production of the free-ends, used to form PL products, with an ideally-free process of DNA fragmentation. In the laboratory, this ideally-free process could be carried out by DNase digestion or mechanical shearing via sonication.

The most notable difference between the methods of Hi-C and the more recent Meta3C, is that after restriction digest, Hi-C employs additional steps leading to the incorporation of biotin tags at each PL junction. This biotinylation permits Hi-C libraries to be subsequently enriched for fragments containing PL junctions by streptavidin-mediated affinity purification. Without enrichment, the simpler Meta3C protocol results in a gross mixture of both WGS and PL read-pairs, where only a small percentage of the total read-pair yield (approx. 1%) will possess PL junctions [23]. The enrichment process within Hi-C, however, is not perfectly efficient and WGS read-pairs are still observed (approx. 10–50% of reads contain a PL product) [23]. DNase Hi-C replaces restriction digest with a non-specific endonuclease (e.g. DNase I) [24] or mechanical DNA shearing process (e.g. sonication) [12]. In this operational mode, Sim3C treats DNA cleavage as a completely unbiased (free) process and as such all genomic positions have equal probability of participating in proximity ligation events.

Within Sim3C, each of the three methodological variations is conceptualised as a sequencing strategy (figure 1) and each iteration of a strategy produces one read-pair (PL or WGS in origin). For all strategies, an iteration begins by drawing a 3-tuple of insert parameters: length, direction and junction point (L_{ins}, dir, x_{junc}).

After obtaining insert parameters, the Hi-C strategy (figure 1a) first tests if the insert will represent a WGS or PL read-pair ($\sim Bern(p_{eff})$), where efficiency p_{eff} is defined in the sense of enrichment. When $p_{eff} = 1$, there is perfect filtering and all WGS read-pairs are eliminated from the experiment. In the case of WGS, the iteration reaches an end-point and the simulation emits a conventional read-pair drawn from the community definition. In the case of PL, a cut-site 3-tuple is drawn (gen_1, chr_1, x_1), where the categorical distribution over chromosomes is weighted by relative abundances (A) and chromosomal copy-numbers (n_{cpy}); genomic position is sampled uniformly from the set of restriction sites $(sites(chr_1))$; and parent genome (gen_1) is implicit from the chromosome. Next, a spurious ligation test is performed ($\sim Bern(p_{spur})$). If a spurious event has occurred, the 3-tuple defining the second cut-site (gen_2, chr_2, x_2) is drawn i.i.d. as the first. If not spurious, next a test for inter-chromosomal (*trans*) ligation is performed. Only source chromosome and position (chr_2, x_2) need be drawn as the second genome is implicitly the same as the first $(gen_2 = gen_1)$. Here, chr_2 is selected without replacement from the set of chromosomes of genome (gen_1), where the categorical distribution is adjusted by removal of chr_1. Finally, an intra-chromosomal (*cis*) ligation must have occurred. As now both genome and chromosome are implicit $(gen_2 = gen_1, chr_2 = chr_1)$, all that is left is to draw genomic position x_2. The pair of positions (x_1, x_2) are constrained by their separation $(s = |x_2 - x_1|)$, which is represented by a mixture model of the geometric and uniform distributions (equation 1). This relation possesses rapid falloff with increasing separation and non-zero probability

for all chromosomal positions, as has been commonly observed in real experimental data [10, 22].

$$Pr(X = s|\alpha, \beta, l) = \beta(1 - \alpha)^s\alpha + (1 - \beta)/l \quad (1)$$

where β is a mixing parameter, α the geometric distribution shape parameter and l chromosome length.

For Meta3C (figure 1b) after insert parameters are determined, in the same fashion as a regular WGS read, an initial free genomic position is drawn (chr_1, x_1^*), uniformly distributed over the extent of chr_1 rather than only over its cut-sites. In real datasets, it has been observed that neither the restriction digestion nor the re-ligation of free ends are perfectly efficient. Taken as independent probabilities, in our model we conceptualise their joint occurrence as an efficiency factor, p_{eff} and a Bernoulli trial $(Bern(p_{eff}))$ determines whether a sequence read is successful in containing an observable proximity ligation event. Failing this coverage test relegates the iteration and end-point and emit a WGS read-pair. Successful candidates instead continue akin to the Hi-C decision tree, beginning with the test for spurious ligation.

For both Hi-C and Meta3C, PL read-pairs are produced by joining the free-ends drawn above as defined by the fragment parameters (figure 2a). Here the location of the PL junction within the insert is determined by x_{junc}. At the junction, Hi-C differs from Meta3C as the process of biotinylation results in the duplication of the restriction cut-site overhang sequence. The overhang duplication in Hi-C is included in the simulation.

DNase Hi-C is handled similarly to traditional Hi-C, with the exception that, as *in-silico* digestion trivially leads to all sites, the simulated digestion is unnecessary to perform and positions can be drawn directly from the uniform distribution over the interval $[0..L_{chr})$. Site duplication, attributable to the likely production of random overhangs in this scenario, is not presently simulated.

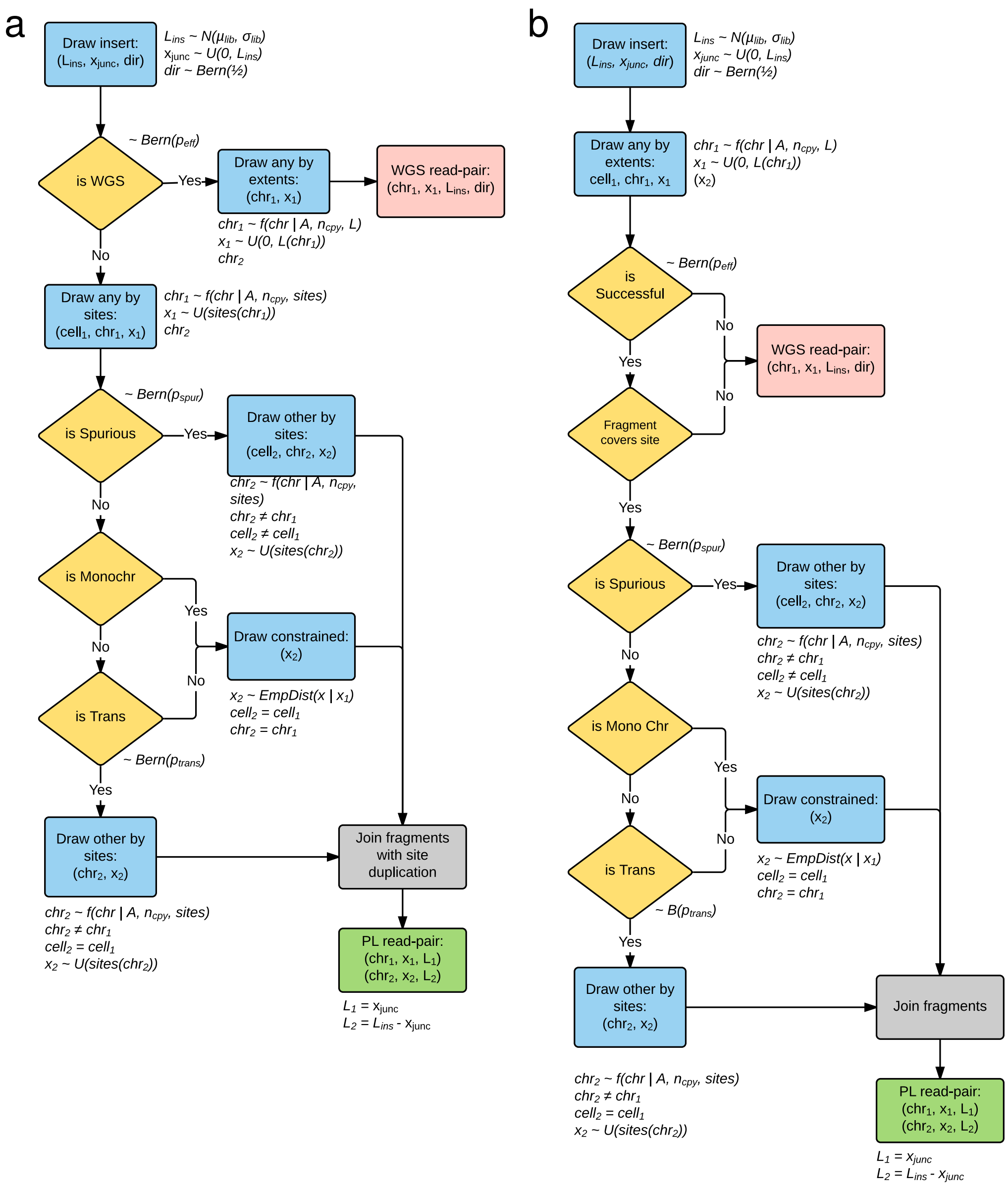

Figure 1. Logical schema used within Sim3C. (**a**) Hi-C and (**b**) Meta3C simulation strategies. Gold diamonds represent simple Bernoulli trials. Blue boxes represent sampling distributions defined by runtime input data (community profile, genomic sequences, enzyme) and the empirically derived distribution for intra-chromosome (*cis*) interaction probability (equation 1). Logical end-points to a single iteration of either algorithm are represented as red (producing a WGS read-pair) and green boxes (producing a PL read-pair). Due to the elimination of the biotinylation step, Meta3C does not produce a duplication of the restriction cut-site overhang (grey boxes).

Structurally related interactions

Independent of any 3D structure that might exist, the primary and most frequently observed interactions are those which occur along a chromosome (intra-arm) (figure 2b), seen as the primary ($y \simeq x$) diagonal in the contact map. Sim3C can approximate the

less frequent interactions occurring between chromosomal arms (inter-arm) [19], which are visible as anti-diagonal ($y \simeq L - x$) in the contact map.

At progressively smaller scales, the hierarchical 3D folding of DNA into topologically associated domains (TADs) produces overlapping regions of interaction visible in the contact map as block-like intensity modulations. Though the agents responsible for their formation vary [1, 3], the characteristic patterns evident in real-data derived 3C contact maps have been observed across all three domains [10, 19, 40]. Sim3C can optionally approximate the sense of TAD related modulation by means of a recursive stochastic process.

Our approximation of hierarchical folding begins from the full extent L of a chromosome (figure 2c). Folding is portrayed by the division of the interval $[0..L)$ into a set of non-overlapping sub-intervals $\{[0, x_1), [x_1, x_2), \cdots, [x_{n-1}, x_n)\}$, the number and widths of which are drawn at random $(U(l_{min}, l_{max}), U(n_{min}, n_{max}))$. The procedure is then recursively applied to each sub-interval until a depth d, producing a nested set of coverings of the full interval $[0..L)$ at progressively finer scales. Across this hierarchical collection each interval is assigned a uniformly distributed random probability p_i and empirical distribution $f_i(s|\theta_i)$ (equation 1) for separation s parameterised by shape parameter α_{TAD} and interval length $l_{inv} = x_{i+1} - x_i$, where $\theta = (\alpha_{TAD}, \beta, l_{inv})$.

The process of drawing samples of separation begins by determining the set of intervals $\{l_{inv}\}$ which contain an initial point x_0. The intervals, as tuples $(p_i, f_i(s|\theta_i))$, then form a categorical distribution (equation (7)), from which a governing distribution $f_i(s|\theta_i)$ is drawn and finally a sample of separation is taken, $s \sim f_i(s|\theta_i)$. To efficiently sample from the full collection, an interval-tree data structure is employed. When queried, an interval-tree returns the set of intervals $\{l\}$ overlapping a position x in order $O(\log n + m)$, where n is number of intervals and m is number of intervals returned by the query.

$$\mathbf{f} = \{f_0(s|\theta_0), f_1(s|\theta_1), \cdots, f_i(s|\theta_i)\} \tag{2}$$

$$N = \text{number of distributions} = |\mathbf{f}| \tag{3}$$

$$\mathbf{p} = \{p_0, p_1, \cdots, p_i\} \tag{4}$$

$$p_i \sim U(0, 1) \text{ and } \sum p_i = 1 \tag{5}$$

$$n \sim Cat(N, \mathbf{p}) \tag{6}$$

$$f(s|n) = \prod_{i=0}^{N-1} f_i(s|\theta_i)^{[i=n]} \tag{7}$$

where $[i = n]$ is the Iverson bracket.

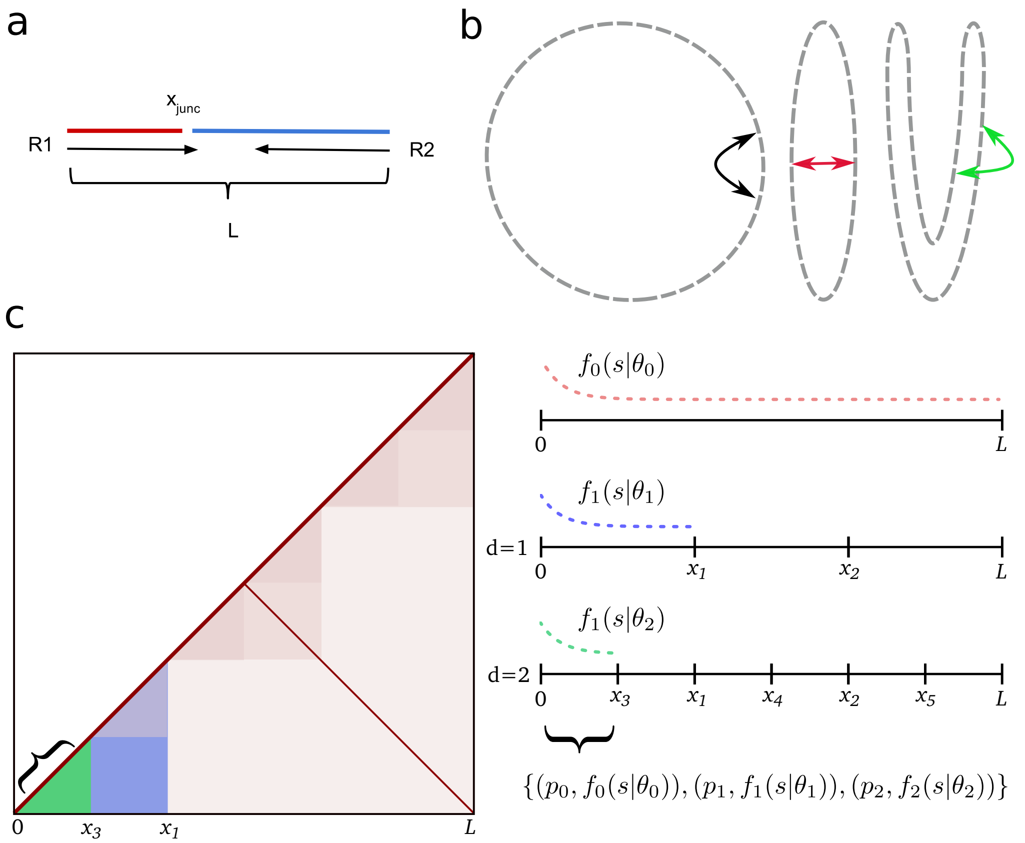

Figure 2. Model details. Generation of proximity ligation inserts (**a**) involves joining two randomly drawn parts (red and blue), from which the read-pair (R1, R2) is then simulated. The junction point (x_{junc}) varies over the interval $[0..L)$ and reproduction of read-through events is possible. For an unbounded chromosome (**b**) (circular here), besides strictly primary separation (black arrow) spatial proximity can be induced from successive folding (red, green arrows). When the spatial arrangement is consistent across the population of cells, this will be observable as modulations in the contact frequencies. Sim3C models simple structurally related modulation of observed contact frequencies (**c**). Beyond primary interactions forming the main diagonal, users can reproduce inter-arm mediated anti-diagonals. Finer scale modulations attributed to topologically associated domains (TADs) can optionally be randomly simulated. Primary interactions $f_0(s|\theta_0)$ (equation 1) cover the full interval $[0, L)$. Each level of recursion ($d = 1, 2 \cdots n$) generates a finer set of intervals, to which a distribution $f_i(s|\theta_i)$ and probability p_i is assigned. The final covering of intervals each define a range (green, curly braces) over which a set of probabilities and empirical distribution pairs govern interaction separation s.

Example scenarios

In the following, three use-cases are presented to demonstrate aspects of the resulting simulation output: bacterial genome, multi-chromosomal eukaryotic (yeast) genome, and metagenome. For each use-case, 3C contact maps have been used to pit simulation output against the corresponding real experimental data (table 1).

Bacterial

A monochromosomal bacterial genome is perhaps the simplest scenario to which proximity ligation methods have been applied, making for a sensible entry point from which to make comparison. Due to the smaller extent, a bright and high resolution contact map (10 kbp bin size) is possible for a practical volume of sequencing data, potentially revealing fine detail not easily discerned with larger bin sizes (50-100 kbp bin size).

The genome of *Caulobacter crescentus* NA1000, a model organism in the study of cellular differentiation and regulation of the cell cycle, is comprised of a single 4 Mbp circular chromosome [28]. Deep Hi-C sequencing of *C. crescentus* has been used to explore the degree to which bacterial chromosomes can be regarded as organised and provided evidence for the existence of so called chromosomal interaction domains (CIDs) [19]. As a prokaryotic analog of topologically associated domains (TADs) from eukaryotic literature [1, 30, 33], these regions are believed to promote intra-domain loci interactions and thereby act to functionally compartmentalize the genome. This chromosomal structure was observed to be at once disruptable through rifampicin mediated inhibition of transcription and malleable by the movement of highly expressed genes [19].

For the raw contact map of *C. crescentus*, prominent rectilinear features are apparent for both real and simulated traditional Hi-C sequencing data (figure 3a,b), while notably for simulated unrestricted Hi-C the field is much smoother (figure 3c). Within the Sim3C model, a single distribution governs both intra- and inter-arm interactions. Inspection of the real-data contact map (figure 3a) suggests that the true relationship governing inter-arm interactions is more dispersed. This perhaps is not surprising, where different arms associating spatially possess a greater number of potential configurations than can be taken on by the primary chromosome backbone. Additionally for the real contact map, long-range interactions away from either diagonal can be seen to drop to a lower threshold than that produced from simulation.

Within the unrestricted Hi-C map, the fine zero-intensity rectilinear features are a direct result of poor mappability (non-unique sequence), where their small size reflects the extent of the non-unique regions (example: rRNA genes) and the single base-pair resolution of the less constrained read generation process. The process of enzymatic digestion is the only difference between the unrestricted and traditional Hi-C simulation models. The clear contrast in their contact maps is thus a combination of factors either directly inherent to digestion (cut-site density) or a byproduct of downstream bioinformatics analysis (e.g. filtering heuristics). Though the problem of mappability exists for any reference based representation, for real and simulated traditional Hi-C, zero-intensity rectilinear features mark regions devoid of cut-sites over at least 10 kbp.

Enabling TAD approximation in simulated traditional Hi-C (figure 3d) has the effect of modulating map intensity in a manner not particularly distinct from that produced purely from experimental/workflow bias. Discriminating between these two feature sources; one representing experimental signal, the other representing noise; demands attention when developing solutions to problems such as normalisation. Contact map normalisation methods, whether based upon explicit or implicit bias models [38], may leave behind remnants of noise-related features from either a lack of convergence or model limitations. Downstream inferencing should therefore not be made under an assumption of bias-free signal.

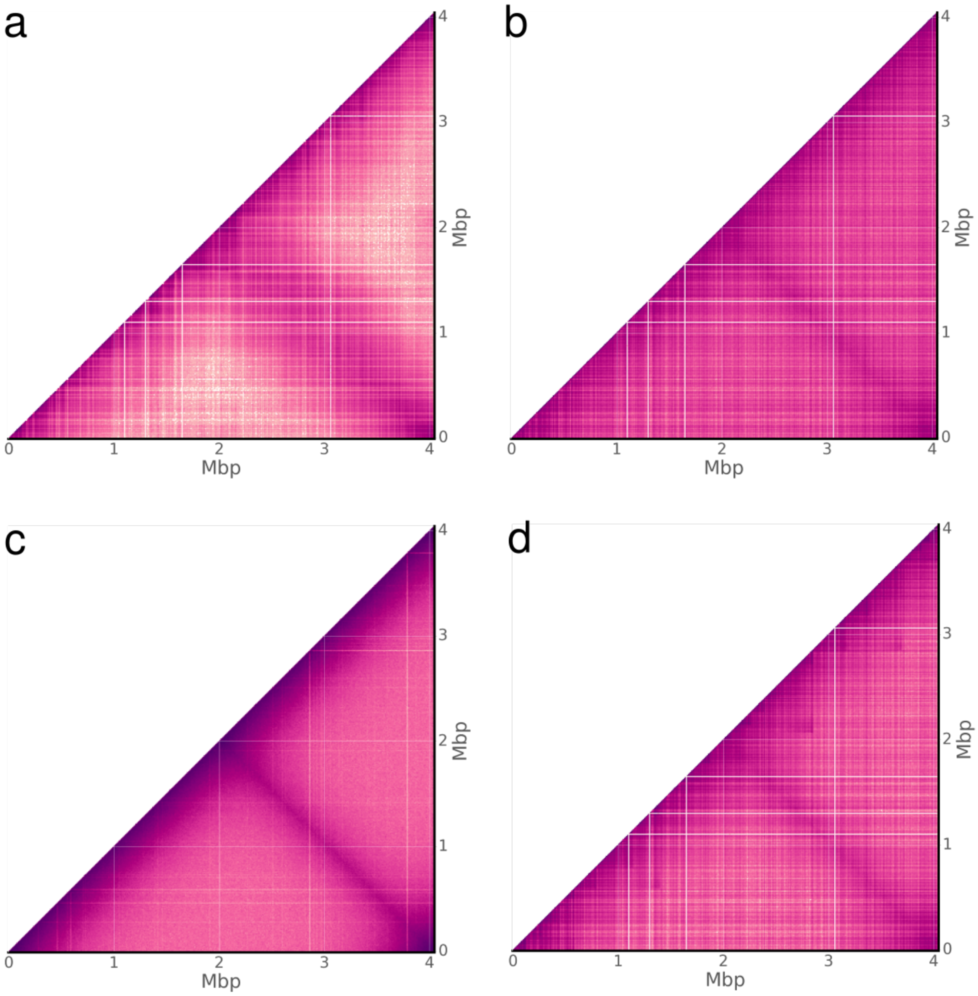

Figure 3. Bacterial contact maps. Observed Hi-C interactions for the monochromosomal genome of *Caulobacter crescentus* NA1000. Comparing (**a**) real experimental data [19], to the three simulation choices (**b**) traditional Hi-C, (**c**) DNase Hi-C and (**d**) traditional Hi-C with TADs enabled. Sharp rectilinear modulations of the intensity within (**a**) and (**b**) indicate a reduction in PL observations within a given bin. Not due to 3D chromosome structure, rather such features can be attributed largely to mappability and low cut-site density. (**c**) Without an enzymatic constraint a significantly smoother field is apparent, yet still susceptible to mappability. (**d**) Enabling topologically associated domains (TADs) highlights the similarity between features produced merely from biases and what could be truly associated with 3D structure.

Eukaryotic

The eight chromosomes of the 15.4 Mbp genome of the native xylose-fermenting yeast *Scheffersomyces stipitis* CBS 6054 [16] range in size from 970 kbp to 3.5 Mbp. The organism was one of 16 yeasts included in a synthetic community to explore the application of Hi-C sequencing to deconvolving metagenomic assemblies [6] and is divergent enough from other synthetic community members to permit unambiguous read mapping, and thus act as a proxy for a clonal experiment.

From the contact map of real Hi-C data (figure 4a), it can be seen that the rates of intra-chromosomal and inter-chromosomal interactions are roughly equivalent in magnitude. Across the eight chromosomes of *S. stipitis*, there is significant uniformity in the degree of physical intimacy within and between all chromosomes. The subtleties of this chromosomal organisation reveals a self-similar “fuzzy-x” pattern repeated between all chromosomes across the contact map. The convergence point within the pattern is attributed to centromere-SPB binding and has been used to predict centromere locations [42]. It has been shown that the physical constraints generated from the interaction of centromeres to the spindle pole body (SPB) and telomeres to the nuclear envelope are sufficient to explain a number of experimental observations in real data [13, 43]. As Sim3C was derived from study of bacterial datasets, our simulation model does not currently include a notion of these higher organism physical constraints. Consequently, the contact map derived from simulated traditional Hi-C sequencing elicits a flat field (figure 4b), where the intensity variation that does exist is a byproduct of aforementioned factors such as mappability and cut-site density. For the runtime parameters employed, the rate of intra-chromosomal contact is higher than that of inter-chromosomal, making clear the boundaries between the eight chromosomes (figure 4b). Though our model is presently incomplete for higher organisms, there remains a potential utility as an analytical or simply observational prior.

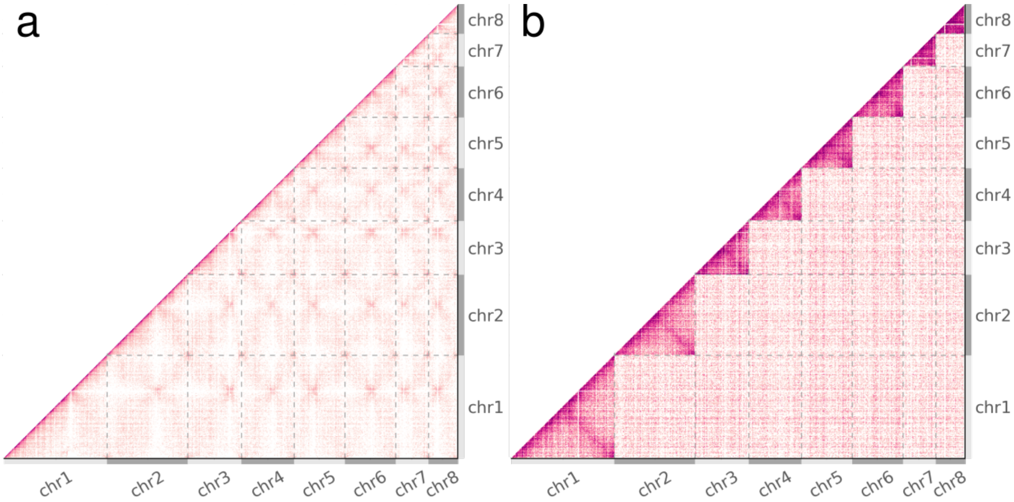

Figure 4. Eukaryotic contact maps. Observed Hi-C interactions (**a**) real and (**b**) simulated data from the eight chromosome genome of the budding yeast *Scheffersomyces stipitis* CBS 6054 [6]. Grey dashed lines and alternating light and dark grey axes demarcate the boundaries between chromosomes. (**b**) Simulated data elicits a flat field and the clearly evident higher rate of intra- to inter- interactions makes for easily observable chromosomal boundaries within the map. (**a**) Contrastingly for real data, the similar rates of intra-chr and inter-chr interactions reveals the physical constraints imposed by centromere-SPB tethering on all eight chromosomes [42].

Metagenomic

In the deconvolution of metagenomes, proximity ligation methods hold great potential as new sources of information and have been investigated by the construction and sequencing of synthetic communities [4, 6, 27]. We selected two previously constructed synthetic bacterial communities, one employing traditional Hi-C and the other Meta3C

(table 1). Intended as "proof of concept" experiments, neither community reflects a real environment, but rather were intended to be easily interpreted and include interesting features, such as: range of GC, single and multi- chromosomal genomes and strain-level divergence. The Hi-C community involved five genotypes from four species, one genome of two chromosomes (*B. thailandensis*), *E. coli* strains BL21 and K12 (Average Nucleotide Identity, ANI 99%) and a wide overall GC range of 37-68% (table 2). Of lower complexity, the Meta3C community involved three genomes from three species, included one genome of two chromosomes (*V. cholerae*) and had a narrower GC range of 44-51% (table 3). Relative to the single genome experiments above, a lower depth of sequencing resulted in a lower overall contact map intensity (figure 5). This is particularly the case for Meta3C, where, by the nature of the method, a large proportion (approx. 99%) of the sequencing yield is in reality conventional WGS read-pair data [27]. As a direct result, in binning the Meta3C dataset, there were insufficient counts to fully establish finer detail within the contact maps, leaving a smoother appearance.

As with single-genome experiments, metagenomic contact maps are locally modulated by factors such as mappability and cut-site density. Importantly now for metagenomes, the factors of relative abundance and GC content interact to alter the observed intensity of each chromosome within the contact map.

As a first approximation and assuming agreement in nucleotide sampling frequency, we expect $n_0 = L/4^{\lambda}$ recognition sites for an enzyme of site length λ and DNA sequence length L. The degree to which an enzyme and DNA sequence deviate from this estimate could be described as how well they match, $m = n_x/n_0$. Poorer quality matches ($m < 1$) occur when an enzyme's recognition site is underrepresented, while conversely, better quality matches ($m > 1$) describe a situation of more recognition sites than expected.

When multiple chromosomes are taken as a community, the relative proportion of sites from each represents an observational bias when conducting 3C-based experiments. For community C, the number of sites n_x from chromosome x determines the number of potential PL pairings N_x within C which involve x (equation 8). The number of intra-chromosomal and inter-chromosomal potential pairs thus respectively vary quadratically and linearly with n_x. Regarding the process of observing a PL event (read-pair) from the community as a random draw with replacement, and the selection pool as comprised of all potential events from all chromosomes, then variation in match quality constitutes a per-chromosome bias. In real laboratory experiments, the composition of the selection pool is further modified by variation in other factors, such as cellular lysis efficiency, unintended DNA fragmentation and relative abundance. In particular, when relative abundances A are introduced, the odds of observing a PL event involving chromosome x is then proportional the product $p_x \propto A_x N_x / N_C$. Although the processes of intra-chromosomal, inter-chromosomal, and inter-cellular (spurious) ligation are treated independently in our simulation model, in this manner, per-chromosome intensity (observation rate of chromosome x) can vary significantly within a metagenome.

$$N_x = n_x^2 + n_x \sum_{n_y \in C \setminus n_x} n_y \tag{8}$$

Though the original laboratory experiments reported by Beitel et al. 2014 and Marbouty et al. 2014 intended o create synthetic communities with uniform relative abundances, in practice each possesses a non-uniform profile. The variation in GC content is largest for the Hi-C experiment and together with non-uniform relative abundances produces a wide range of chromosome intensity for both real and simulated data (figure 5a,b). For both the real and simulated Hi-C maps, the frequent observation of PL events involving *P. pentosaceus* (Pp) and *L. brevis* (Lb), suggests the possibility

that inter-cellular interaction is significant. Within the simulated map at least, inter-cellular pairs are produced exclusively through the process of spurious ligation (noise) and are observed at a higher rate than in the real data, indicating that as expected, spurious ligation rates across species are correlated with their relative abundances.

Further for the Hi-C data, the two-chromosome genome of *B. thailandensis* (Bt1, Bt2) (figure 5a) has a greater rate of inter-chromosomal interaction than expected from comparing it to simulation (figure 5b). Meanwhile, the clear delineation of *E. coli* strains BL21 and K12 ($ANI > 99\%$), with little inter-cellular signal, helps to support the notion that the inter-chromosomal interactions observed between *B. thailandensis* chromosomes ($ANI \simeq 83\%$) are real and not a by-product of inadequate filtering.

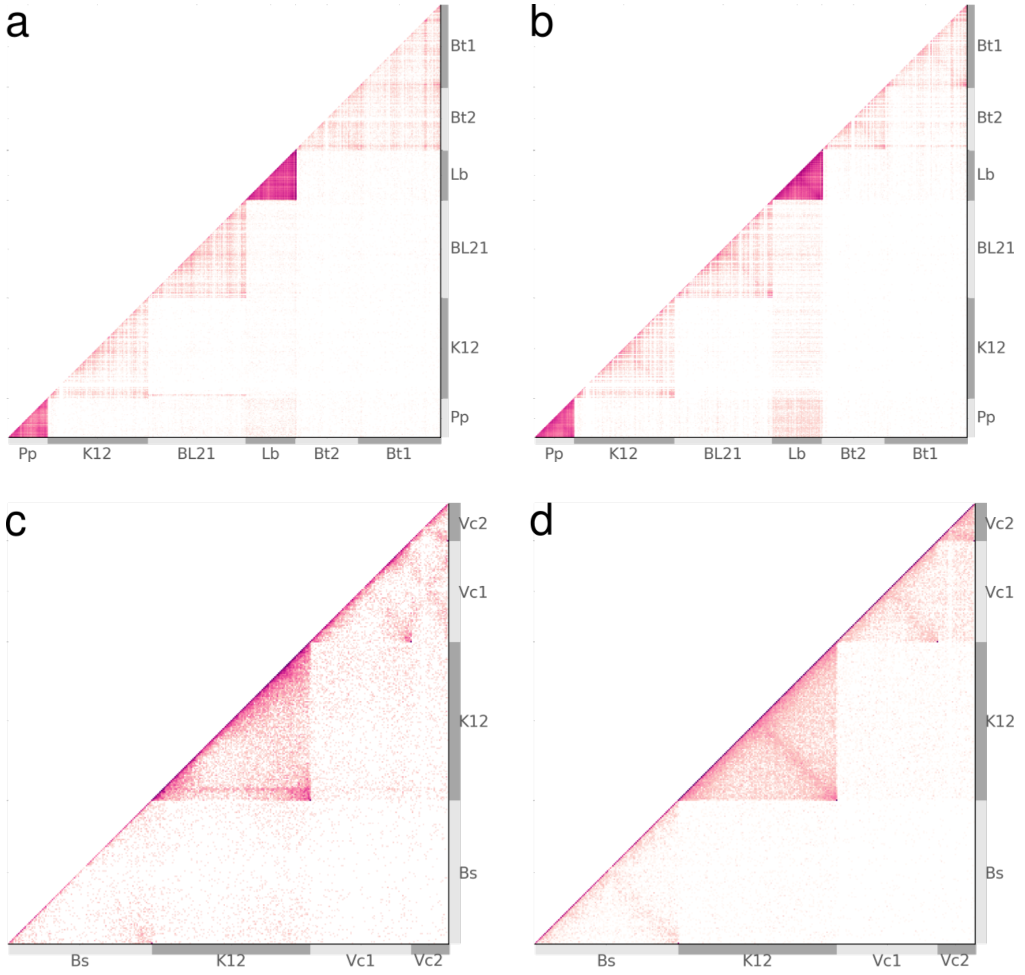

Figure 5. Metagenomic contact maps. From synthetic microbial communities, raw contact maps from real (**a**) and simulated (**b**) traditional HiC, and real (**c**) and simulated (**d**) Meta3C. Chromosome boundaries are demarcated by alternating light and dark grey bands (tables 2, 3), while the small plasmids of *L. brevis* are omitted for clarity. Although the original works [4, 27] intended uniform abundance, the results exhibit significant variation in abundance. Lysis efficiency (not modelled) and enzyme suitability are significant factors contributing to the overall intensity of a given chromosome. For more abundant members of the Hi-C community (*P. pentosaceus* and *L. brevis*), signal due only to spurious ligation can appear to suggest inter-cellular interactions when none are present (**b**).

Limitations and future work

Sim3C in its current form has several limitations, some of which present opportunities for future work. Sim3C's repertoire of structural features is currently limited to those found in microbes - circular and linear chromosomes with randomly generated approximations of self-associating domains (CIDs/TADs). Sim3C does not model structural features observed in larger, more complex genomes (CTCF/cohesin loops, A/B compartments, chromosome territories) [22, 36]. Such features are becoming increasingly well characterised [41] and a simulator capable of modelling these features would surely be valuable. Mammalian genomes are much larger than microbial genomes

Authors	Type	Method	Accession	Sequencing details	Mapped reads
Beitel et al [4]	Synthetic bacterial metagenome	Hi-C	SRX377733	MiSeq 160bp PE<br>insert range: 280-420bp<br>enzyme: HindIII	20552775
Burton et al [6]	Synthetic yeast metagenome	Hi-C	SRX527868	HiSeq2500 100bp PE<br>insert range: 450-550bp<br>enzyme: HindIII	9704944
Le et al [19]	Single bacterial genome	Hi-C	SRX263925	HiSeq2000 40bp PE<br>insert range: 200-600bp<br>enzyme: NcoI	22324360
Marbouty et al [26]	Synthetic bacterial metagenome	Meta3C	doi:10.5061/ dryad.gv595	HiSeq2000 100bp PE<br>insert range: 400-800bp<br>enzyme: HpaII	7975740

Table 1. Real Hi-C and Meta3C data-sets used within this work. The total off-diagonal weight of the contact map was used to calibrate the amount of simulated sequencing required to approximately match the outcome of the real experiments.

however, and additional work to improve scalability of Sim3C will likely be required.

Some features of microbial eukaryotes, such as the point centromeres found in budding yeast genomes [7] are computationally simpler [13, 42] yet remain unmodelled in Sim3C. The addition of these sorts of model details would be best supported by introducing model initialisation via external data (experimental observations, motif detection, cell phase), which subsequently would require extension of the community profile definition. Careful design would be required to ensure these features could be added without compromising ease-of-use.

Methods

Reference Data

To compare Sim3C against real experiments, we obtained previously published experimental read-pair datasets (table 1) and their accompanying reference genomes (tables 2, 3) from public archives. In the case of the single genome project of *Caulobacter crescentus* CB15 [19], sequencing data derived from untreated swarmer cells was chosen and the laboratory strain *C. crescentus* NA1000 (acc: NC_011916) was used as the reference genome. For the yeast genome, the completed eight chromosome genome of *Scheffersomyces stipitis* CBS 6054 was used as a reference (acc: PRJNA18881) and the respective reads were extracted from the MY16 yeast synthetic metagenome [6] by direct mapping with BWA MEM. Extraction by mapping in isolation was employed as *S. stipitis* was the second furthest phylogenetically removed yeast in the synthetic community and was the most contiguous (N50: 60kbp) from the whole synthetic community de novo metagenomic WGS assembly.

Read Generation

Experimental parameters used in read simulation were set to agree as closely as reasonably possible to the respective real experiments, employing the same read length and restriction enzyme (table 1). In each experiment, the published fragment size range was approximated by a normal distribution (table 4). For ease of reproducibility, a

Name	Replicons	Accession	Chr abbr.	A	n_{cpy}	%GC	n_x	m
Burkholderia thailandensis E264	2	NC_007651<br>NC_007650	Bt1<br>Bt2	0.054	1	67.29<br>68.07	225<br>144	0.24<br>0.20
Escherichia coli BL21	1	NC_012892	BL21	0.242	1	50.83	508	0.46
Escherichia coli K12 DH10B	1	NC_010473	K12	0.166	1	50.78	568	0.50
Lactobacillus brevis ATCC 367	3	NC_008497<br>NC_008498<br>NC_008499	Lb<br>-<br>-	0.436	1	46.22<br>38.64<br>38.51	629<br>3<br>16	1.12<br>0.92<br>1.84
Pediococcus pentosaceus ATCC 25745	1	NC_008525	Pp	0.102	1	37.36	863	1.93

Table 2. Synthetic Hi-C community. A synthetic community used to demonstrate the utility of Hi-C sequencing data in resolving a microbial metagenome [4]. It is composed of 5 bacteria, including two closely related strains (*E. coli* K12 and BL21), a genome with two plasmids (*L. brevis*) and a two-chromosome genome (*B. thailandensis*). A is relative abundance, n_{cpy} is copy number, n_x is number of restriction sites, and $m = n_x/n_0$ is match quality between chromosome and enzyme choice: $m < 1$ is worse, $m > 1$ is better.

Name	Replicons	Accession	Chr abbr.	A	n_{cpy}	%GC	n_x	m
Bacillus subtilis subsp. subtilis str. 168	1	NC_000964	Bs	0.123	1	43.51	14529	0.88
Escherichia coli str. K-12 substr. MG1655	1	NC_000913	K12	0.562	1	50.79	24311	1.34
Vibrio cholerae O1 biovar El Tor str. N16961	2	NC_002505<br>NC_002506	Vc1<br>Vc2	0.332	1	47.70<br>46.91	5909<br>1802	0.51<br>0.43

Table 3. Synthetic Meta3C community. A synthetic community used to demonstrate the utility of Meta3C sequencing data in resolving a microbial metagenome [26,27]. It is composed of three bacteria with one possessing two chromosomes. A is relative abundance, n_{cpy} is copy number, n_x is number of restriction sites, and $m = n_x/n_0$ is match quality between chromosome and enzyme choice: $m < 1$ is worse, $m > 1$ is better.

Experiment	Insert μ (bp)	Insert σ (bp)	Anti rate	Spurious rate	Trans rate	Reads ($\times 10^6$)
Beitel et al	300	50	0.2	0.05	0.1	7
Burton et al	400	50	0.2	0.5	0.15	1.5
Le et al	400	100	0.2	0.2	0.1	22
Marbouty et al	600	100	0.2	0.2	0.2	7.5

Table 4. Runtime simulation. Parameters supplied to Sim3C during read generation.

single random seed (1234) was used in all simulations. As our intent was primarily to demonstrate functionality, rates of inter-chromosomal and spurious events were adjusted per-experiment only through a qualitative process. For simulation of metagenomic datasets, relative abundances were estimated by mapping real experimental reads to the respective reference genomes. From each real experiment, the off-diagonal weight of the resulting contact map was used to calibrate the amount of simulated sequencing required to achieve roughly equivalent intensity (table 4). Both real and simulated read-pair datasets were mapped to their respective reference genomes using BWA MEM (v0.7.15-r1140, RRID:SCR_010910) [21]

Contact Maps

Contact maps were produced using our own tool (`contact_map.py`), where heatmap intensity was plotted as log-scaled observational frequency. All aligned reads were subject to the same basic filtering criteria: BWA MEM mapq > 5 and alignment length $\geq 50\%$ of read length, with the added restriction that read alignments must have begun with a match. For methods which employed a restriction enzyme (traditional Hi-C, Meta3C), we constrained the maximum allowable distance from an aligned read to the nearest upstream cut-site. Calculated per chromosome, this distance constraint could not exceed two-fold the median cut-site spacing. Rather than simply delete the primary diagonal for the sake of reducing the displayed dynamic range in figures, we instead to reduced its intensity by categorizing properly paired reads with an estimated fragment size of less than 2 of the reported mean as being conventional WGS (non-PL) reads and ignored them. The resolution of contact maps was adjusted between experiments so as to present a sufficiently bright image without undue loss of resolution. The contact map bin sizes employed were: 10000 bp for the single bacterial genome, 25000 bp for the yeast genome and 40000 bp for the Hi-C and Meta3C metagenomes (tables 2, 3).

Availability of data and materials

Snapshots of the supporting code are available from the GigaScience repository, GigaDB [32].

Availability of supporting source code and requirements

- Project name: Sim3C
- Release version: 0.1
- Project homepage: https://github.com/cerebis/sim3C
- RRID: SCR_015772
- DOI: 10.5281/zenodo.1030812
- Operating system: Platform independent
- Programming languages: Python 2.7
- License: GNU GPL v3

Declarations

List of abbreviations

- IPC - interprocess communication
- PL - proximity ligation
- WGS - whole genome shotgun
- CID - chromosomal interaction domain
- TAD - topologically associated domain
- $Bern(x)$ - Bernoulli distribution
- $U(x)$ - uniform distribution
- $N(\mu, \sigma)$ - normal distribution
- *cis* - intra-chromosomal
- *trans* - inter-chromosomal

Ethics approval and consent to participate

Not applicable

Competing interests

The authors declare that they have no competing interests.

Funding

This work was supported under Australian Research Council's Discovery Projects funding scheme (project number: LP150100912, CI: Djordjevic, SP). The NeCTAR Research Cloud is an Australian Government project conducted as part of the Super Science initiative and financed by the and the Education Investment Fund (EIF) and National Collaborative Research Infrastructure Strategy (NCRIS).

- https://www.education.gov.au/education-investment-fund
- https://www.education.gov.au/national-collaborative-research-infrastructure-strategy-ncris

Authors contributions

MD designed and implemented Sim3C and wrote the manuscript and prepared figures. AD assisted in the design and contributed to the manuscript.

Acknowledgements

We thank Steven P. Djordjevic for his support and helpful discussions. This work was supported by the AusGEM initiative, a collaboration between the NSW Department of Primary Industries and the ithree institute. We acknowledge the use of computing resources from the NeCTAR Research Cloud, the QCIF and the UTS eResearch Group.

- http://www.nectar.org.au
- http://www.qcif.edu.au
- https://eresearch.uts.edu.au

References

1. R. D. Acemel, I. Maeso, and J. L. Gómez-Skarmeta. Topologically associated domains: a successful scaffold for the evolution of gene regulation in animals. *Wiley Interdiscip. Rev. Dev. Biol.*, 2 Mar. 2017.

2. F. E. Angly, D. Willner, F. Rohwer, P. Hugenholtz, and G. W. Tyson. Grinder: a versatile amplicon and shotgun sequence simulator. *Nucleic Acids Res.*, 40(12):e94–e94, 1 July 2012.

3. A. Badrinarayanan, T. B. K. Le, and M. T. Laub. Bacterial chromosome organization and segregation. *Annu. Rev. Cell Dev. Biol.*, 31(1):171–199, 1 Jan. 2015.

4. C. W. Beitel, J. M. Lang, I. F. Korf, R. W. Michelmore, J. A. Eisen, and A. E. Darling. Strain- and plasmid-level deconvolution of a synthetic metagenome by sequencing proximity ligation products. *PeerJ*, 2(12):e415, 1 Jan. 2014.

5. J. N. Burton, A. Adey, R. P. Patwardhan, R. Qiu, J. O. Kitzman, and J. Shendure. Chromosome-scale scaffolding of de novo genome assemblies based on chromatin interactions. *Nat. Biotechnol.*, 31(12):1119–1125, 3 Nov. 2013.

6. J. N. Burton, I. Liachko, M. J. Dunham, and J. Shendure. Species-Level deconvolution of metagenome assemblies with Hi-C-Based contact probability maps. *G3*, 4(7):1339–1346, 22 May 2014.

7. G. Cottarel, J. H. Shero, P. Hieter, and J. H. Hegemann. A 125-base-pair CEN6 DNA fragment is sufficient for complete meiotic and mitotic centromere functions in saccharomyces cerevisiae. *Mol. Cell. Biol.*, 9(8):3342–3349, Aug. 1989.

8. J. Dekker, K. Rippe, M. Dekker, and N. Kleckner. Capturing chromosome conformation. *Science*, 295(5558):1306–1311, 15 Feb. 2002.

9. M. Z. DeMaere and A. E. Darling. Deconvoluting simulated metagenomes: the performance of hard- and soft- clustering algorithms applied to metagenomic chromosome conformation capture (3c). *PeerJ*, 4(4):e2676, 1 Jan. 2016.

10. J. R. Dixon, S. Selvaraj, F. Yue, A. Kim, Y. Li, Y. Shen, M. Hu, J. S. Liu, and B. Ren. Topological domains in mammalian genomes identified by analysis of chromatin interactions. *Nature*, 485(7398):376–380, 11 Apr. 2012.

11. O. Dudchenko, S. S. Batra, A. D. Omer, S. K. Nyquist, M. Hoeger, N. C. Durand, M. S. Shamim, I. Machol, E. S. Lander, A. P. Aiden, and E. L. Aiden. De novo assembly of the aedes aegypti genome using Hi-C yields chromosome-length scaffolds. *Science*, 356(6333):92–95, 7 Apr. 2017.

12. M. Fullwood, P. Y. H. Huang, Y. Han, L. Handoko, S. Velkov, E. Wong, E. Cheung, X. Ruan, C.-L. Wei, M. J. Fullwood, and Y. Ruan. Protocol: Sonication-based circular chromosome conformation capture with next-generation sequencing analysis for the detection of chromatin interactions. *Protocol Exchange*, 14 Dec. 2010.

13. K. Gong, H. Tjong, X. J. Zhou, and F. Alber. Comparative 3D genome structure analysis of the fission and the budding yeast. *PLoS One*, 10(3):e0119672, 23 Mar. 2015.

14. X. Hu, J. Yuan, Y. Shi, J. Lu, B. Liu, Z. Li, Y. Chen, D. Mu, H. Zhang, N. Li, Z. Yue, F. Bai, H. Li, and W. Fan. pIRS: Profile-based illumina pair-end reads simulator. *Bioinformatics*, 28(11):1533–1535, 1 June 2012.

15. W. Huang, L. Li, J. R. Myers, and G. T. Marth. ART: a next-generation sequencing read simulator. *Bioinformatics*, 28(4):593–594, 15 Feb. 2012.

16. T. W. Jeffries, I. V. Grigoriev, J. Grimwood, J. M. Laplaza, A. Aerts, A. Salamov, J. Schmutz, E. Lindquist, P. Dehal, H. Shapiro, Y.-S. Jin, V. Passoth, and P. M. Richardson. Genome sequence of the lignocellulose-bioconverting and xylose-fermenting yeast pichia stipitis. *Nat. Biotechnol.*, 25(3):319–326, Mar. 2007.

17. B. Jia, L. Xuan, K. Cai, Z. Hu, L. Ma, and C. Wei. NeSSM: a next-generation sequencing simulator for metagenomics. *PLoS One*, 8(10):e75448, 1 Jan. 2013.

18. J. O. Korbel and C. Lee. Genome assembly and haplotyping with Hi-C. *Nat. Biotechnol.*, 31(12):1099–1101, Dec. 2013.

19. T. B. K. Le, M. V. Imakaev, L. A. Mirny, and M. T. Laub. High-resolution mapping of the spatial organization of a bacterial chromosome. *Science*, 342(6159):731–734, 8 Nov. 2013.

20. H. Li. lh3/wgsim. `https://github.com/lh3/wgsim`, 18 Oct. 2011. Accessed: 2017-3-21.

21. H. Li. Aligning sequence reads, clone sequences and assembly contigs with BWA-MEM. *arXiv.org*, q-bio.GN, 17 Mar. 2013.

22. E. Lieberman-Aiden, N. L. van Berkum, L. Williams, M. Imakaev, T. Ragoczy, A. Telling, I. Amit, B. R. Lajoie, P. J. Sabo, M. O. Dorschner, R. Sandstrom, B. Bernstein, M. A. Bender, M. Groudine, A. Gnirke, J. Stamatoyannopoulos, L. A. Mirny, E. S. Lander, and J. Dekker. Comprehensive mapping of long-range interactions reveals folding principles of the human genome. *Science*, 326(5950):289–293, 9 Oct. 2009.

23. M. Liu and A. Darling. Metagenomic chromosome conformation capture (3c): techniques, applications, and challenges. *F1000Res.*, 4(1377):1–9, 1 Jan. 2015.

24. W. Ma, F. Ay, C. Lee, G. Gulsoy, X. Deng, S. Cook, J. Hesson, C. Cavanaugh, C. B. Ware, A. Krumm, J. Shendure, C. A. Blau, C. M. Disteche, W. S. Noble, and Z. Duan. Fine-scale chromatin interaction maps reveal the cis-regulatory landscape of human lincRNA genes. *Nat. Methods*, 12(1):71–78, 1 Jan. 2015.

25. M. Marbouty, L. Baudry, A. Cournac, and R. Koszul. Scaffolding bacterial genomes and probing host-virus interactions in gut microbiome by proximity ligation (chromosome capture) assay. *Sci Adv*, 3(2):e1602105, Feb. 2017.

26. M. Marbouty, A. Cournac, J.-F. Flot, H. Marie-Nelly, J. Mozziconacci, and R. Koszul. Data from: Metagenomic chromosome conformation capture (meta3c) unveils the diversity of chromosome organization in microorganisms. 2014.

27. M. Marbouty, A. Cournac, J.-F. Flot, H. Marie-Nelly, J. Mozziconacci, and R. Koszul. Metagenomic chromosome conformation capture (meta3c) unveils the diversity of chromosome organization in microorganisms. *Elife*, 3(e03318):e03318, 17 Dec. 2014.

28. M. E. Marks, C. M. Castro-Rojas, C. Teiling, L. Du, V. Kapatral, T. L. Walunas, and S. Crosson. The genetic basis of laboratory adaptation in caulobacter crescentus. *J. Bacteriol.*, 192(14):3678–3688, July 2010.

29. T. Nagano, C. Várnai, S. Schoenfelder, B.-M. Javierre, S. W. Wingett, and P. Fraser. Comparison of Hi-C results using in-solution versus in-nucleus ligation. *Genome Biol.*, 16:175, 26 Aug. 2015.

30. E. P. Nora, B. R. Lajoie, E. G. Schulz, L. Giorgetti, I. Okamoto, N. Servant, T. Piolot, N. L. van Berkum, J. Meisig, J. Sedat, J. Gribnau, E. Barillot, N. Blüthgen, J. Dekker, and E. Heard. Spatial partitioning of the regulatory landscape of the x-inactivation centre. *Nature*, 485(7398):381–385, 17 May 2012.

31. Y. Ono, K. Asai, and M. Hamada. PBSIM: PacBio reads simulator–toward accurate genome assembly. *Bioinformatics*, 29(1):119–121, 1 Jan. 2013.

32. B. S. Pedersen, R. L. Collins, M. E. Talkowski, and A. R. Quinlan. Supporting software for “indexcov: fast coverage quality control for whole-genome sequencing”, 2017.

33. B. D. Pope, T. Ryba, V. Dileep, F. Yue, W. Wu, O. Denas, D. L. Vera, Y. Wang, R. S. Hansen, T. K. Canfield, R. E. Thurman, Y. Cheng, G. Gulsoy, J. H. Dennis, M. P. Snyder, J. A. Stamatoyannopoulos, J. Taylor, R. C. Hardison, T. Kahveci, B. Ren, and D. M. Gilbert. Topologically associating domains are stable units of replication-timing regulation. *Nature*, 515(7527):402–405, 20 Nov. 2014.

34. V. Ramani, D. A. Cusanovich, R. J. Hause, W. Ma, R. Qiu, X. Deng, C. A. Blau, C. M. Disteche, W. S. Noble, J. Shendure, and Z. Duan. Mapping 3D genome architecture through in situ DNase Hi-C. *Nat. Protoc.*, 11(11):2104–2121, 1 Nov. 2016.

35. V. Ramani, X. Deng, R. Qiu, K. L. Gunderson, F. J. Steemers, C. M. Disteche, W. S. Noble, Z. Duan, and J. Shendure. Massively multiplex single-cell Hi-C. *Nat. Methods*, 14(3):263–266, 30 Jan. 2017.

36. S. S. P. Rao, M. H. Huntley, N. C. Durand, E. K. Stamenova, I. D. Bochkov, J. T. Robinson, A. L. Sanborn, I. Machol, A. D. Omer, E. S. Lander, and E. L. Aiden. A 3D map of the human genome at kilobase resolution reveals principles of chromatin looping. *Cell*, 159(7):1665–1680.

37. D. C. Richter, F. Ott, A. F. Auch, R. Schmid, and D. H. Huson. MetaSim: a sequencing simulator for genomics and metagenomics. *PLoS One*, 3(10):e3373, 8 Oct. 2008.

38. A. D. Schmitt, M. Hu, and B. Ren. Genome-wide mapping and analysis of chromosome architecture. *Nat. Rev. Mol. Cell Biol.*, 17(12):743–755, 1 Dec. 2016.

39. S. Selvaraj, J. R Dixon, V. Bansal, and B. Ren. Whole-genome haplotype reconstruction using proximity-ligation and shotgun sequencing. *Nat. Biotechnol.*, 31(12):1111–1118, Dec. 2013.

40. T. Sexton, E. Yaffe, E. Kenigsberg, F. Bantignies, B. Leblanc, M. Hoichman, H. Parrinello, A. Tanay, and G. Cavalli. Three-dimensional folding and functional organization principles of the drosophila genome. *Cell*, 148(3):458–472, 3 Feb. 2012.

41. T. J. Stevens, D. Lando, S. Basu, L. P. Atkinson, Y. Cao, S. F. Lee, M. Leeb, K. J. Wohlfahrt, W. Boucher, A. O'Shaughnessy-Kirwan, J. Cramard, A. J. Faure, M. Ralser, E. Blanco, L. Morey, M. Sansó, M. G. S. Palayret, B. Lehner, L. Di Croce, A. Wutz, B. Hendrich, D. Klenerman, and E. D. Laue. 3D structures of individual mammalian genomes studied by single-cell Hi-C. *Nature*, 544(7648):59–64, 6 Apr. 2017.

42. N. Varoquaux, I. Liachko, F. Ay, J. N. Burton, J. Shendure, M. J. Dunham, J.-P. Vert, and W. S. Noble. Accurate identification of centromere locations in yeast genomes using Hi-C. *Nucleic Acids Res.*, 43(11):5331–5339, 23 June 2015.

43. H. Wong, H. Marie-Nelly, S. Herbert, P. Carrivain, H. Blanc, R. Koszul, E. Fabre, and C. Zimmer. A predictive computational model of the dynamic 3D interphase yeast nucleus. *Curr. Biol.*, 22(20):1881–1890, 23 Oct. 2012.

latex source for manuscript Click here to download Manuscript main.tex

Sim3C: simulation of Hi-C and Meta3C proximity ligation sequencing technologies

Matthew Z DeMaere[1], Aaron E Darling[1*],

1 The ithree institute, University of Technology Sydney (Sydney, NSW, Australia)

*** aaron.darling@uts.edu.au**

Abstract

Background

Chromosome conformation capture (3C) and Hi-C DNA sequencing methods have rapidly advanced our understanding of the spatial organization of genomes and metagenomes. Many variants of these protocols have been developed, each with their own strengths. Currently there is no systematic means for simulating sequence data from this family of sequencing protocols, potentially hindering the advancement of algorithms to exploit this new datatype.

Findings

We describe a computational simulator that, given simple parameters and reference genome sequences, will simulate Hi-C sequencing on those sequences. The simulator models the basic spatial structure in genomes that is commonly observed in Hi-C and 3C datasets, including the distance-decay relationship in proximity ligation, differences in the frequency of interaction within and across chromosomes, and the structure imposed by cells. A means to model the 3D structure of randomly generated topologically associating domains (TADs) is provided. The simulator considers several sources of error common to 3C and Hi-C library preparation and sequencing methods, including spurious proximity ligation events and sequencing error.

Conclusions

We have introduced the first comprehensive simulator for 3C and Hi-C sequencing protocols. We expect the simulator to have use in testing of Hi-C data analysis algorithms, as well as more general value for experimental design, where questions such as the required depth of sequencing, enzyme choice, and other decisions can be made in advance in order to ensure adequate statistical power with respect to experimental hypothesis testing.

Keywords

Hi-C, Meta3C, 3C, DNA sequencing, simulation, metagenomics

Findings

Software testing

To the casual observer, formal software testing is often thought to begin and end with the validation of fine-grained behavioural (functional) aspects; such as the correct execution of individual methods. In day to day use however, what can matter most to end-users are broader system attributes such as speed, scalability, reproducibility and ease of use. To ensure a project offers maximum value, a thorough testing process would collectively examine all aspects.

For inferential software within scientific fields, the system-level attributes of precision and accuracy are of primary interest, and their quantification is best accomplished by comparison to a known truth (gold standard). Therefore, any testing methodology capable of providing an *a priori* gold standard, particularly without estimation, improves this facet of testing significantly.

Purpose-built bioinformatics software ultimately acts on experimentally collected observations. The inherent noise and variation that comes with experimental data means achieving testing thoroughness is a great challenge. Ready access to sufficient data sources is a fundamental necessity for adequate software testing.

For established experimental methods, public data archives are a first choice for the necessary testing data. When high quality metadata is available, testing driven by real data becomes possible. However, even when sufficient depth and description of data is available, difficulty can remain in matching desired test data characteristics to what actually exists in one or several public dataset(s). Further, fine-grained whole-corpus querying of metadata on remote data archives is not always possible, frequently making the up-front job of data selection a difficult task. Once selected, obtaining said real data can be time-consuming or even infeasible in locations with lower network speeds and/or high bandwidth costs. In advancing fields such as DNA sequencing, new experimental datatypes can appear for which the public data archives contain only a handful of examples and few researchers would have the time and financial resources to commit to experimental generation of new data purely for software testing.

Though performance on real data is the ultimate arbiter of analytical value, advantaged by explicit control over its characteristics, a faithful simulation of real data can act as a valuable proxy. Simulation-driven development and testing has proven to be a highly cost effective and time efficient approach. It offers the possibility to explore a near continuum of data characteristics, subjecting software to an otherwise unavailable degree of testing thoroughness. Certainty and control makes attaining the twin objectives of rigorous testing and an *a priori* gold standard straightforward. This enables us not only to be more certain about when we have failed, but also to extrapolate this process to infer the limits of success within the experimental parameter space.

Tools for simulating DNA sequencing reads have existed from the very early days of genomics, beginning with the many anonymous implementations of simple DNA shearing algorithms, up to the most recent highly detailed empirical model simulators [14, 15, 20, 31]. From read simulation in isolation, field advancements such as metagenomics have been accompanied soon after by simulators reflecting their specific data characteristics and evolving experimental methodology [2, 17, 37].

We introduce Sim3C, a software package designed to simulate data generated by Hi-C and other 3C-based proximity ligation (PL) sequencing protocols. The software includes flexible support for a range of sequencing project scenarios and choice of three 3C methods (Hi-C, Meta3C, DNase Hi-C). The resulting output (paired-end FastQ) is easily assimilated into existing analysis workflows. It is our intention that Sim3C provide the Hi-C/3C research community with means to further validate existing

software projects, to support new experimental or analysis development initiatives and as a platform for exploration, such as the comparative analysis of clustering algorithms [9].

3C sequencing

3C-based sequencing protocols, including Hi-C, 4C-seq, and Meta3C, have great potential to address questions directed at the spatial organization of DNA in samples ranging from eukaryotic tissue, to single cells, to microbial communities. The growing use of these protocols creates a legitimate need for a simulator capable of generating data with relevant characteristics.

Chromosome conformation capture (3C) was originally designed as a PCR-based assay to measure interactions among a small number of defined regions of eukaryotic chromosomes [8]. In 2009 Lieberman-Aiden [22] reported an extension of the protocol to high throughput sequencing, enabling the global spatial arrangement of chromosomes to be reconstructed at unprecedented resolution. All 3C protocols depend on an initial formalin fixation step, which crosslinks proteins bound to DNA in vivo. Subsequently cells are lysed and the DNA:protein complexes are sheared enzymatically and/or physically to create free ends in the bound DNA strands. These free ends are then subjected to a proximity ligation reaction, in which ligation of free ends preferentially occurs among DNA strands cobound in a protein complex. The DNA:protein crosslinks are then reversed, the DNA is purified, and an Illumina-compatible sequencing library is constructed. In Hi-C protocols, the proximity ligation junctions can then be further purified in the sequencing library.

3C-derived methods have found several applications beyond their initial use to reconstruct 3D chromosome structure. For example, it has been shown that 3C-derived data provide a valuable signal for genome scaffolding [5, 11], as well as a signal that can support genome-wide haplotype phasing [18, 39]. 3C-derived data has also proven valuable for metagenomics, where initial studies on mock communities demonstrated that highly accurate genome reconstruction in mixed microbial communities could be facilitated by proximity ligation sequence data [4, 6, 27]. Subsequent application to naturally occurring microbial communities has also suggested that bacteriophage can be linked to their hosts with this data type [25].

In the remainder of this manuscript we describe the Sim3C software and demonstrate how it can be used to simulate data for various 3C-derived experiments.

Experiment scenarios

Beyond simple monochromosomal genome sequencing experiments, Sim3C offers support for the more complex scenarios of multi-chromosomal genomes and metagenomes. A scenario is defined by way of a community profile; assigning a copy-number and containing genome to each chromosome and a relative abundance to each genome. The profile and supporting reference sequences form a skeleton definition with which to initialize the weighted random sampling process within a simulation. The user can elect to supply a profile either as an explicit table (listing 1, 2) or allow Sim3C to draw abundances at runtime from one of three distributions (equal abundance, uniformly random, log-normal distribution) for communities made up of strictly mono-chromosomal genomes.

```
#chrom  cell  abund  copynum
chr1    bac1  0.4    1
plas1   bac1  0.4    1
chr2    bac2  0.6    1
```

Listing 1. A mock two genome community. For demonstration purposes, we assume that the plasmid (plas1) is present in four copies and that there is a 0.4/0.6 relative abundance split between the two organisms (bac1, bac2) in the community

```
#chrom  cell  abund  copynum
chr1    euk1  1      1
chr2    euk1  1      1
chr3    euk1  1      1
chr4    euk1  1      2
```

Listing 2. A mock four chromosome genome. Cellular abundance is a constant across the profile, while chr4 exists in two copies. Note that relative abundances specified in a profile are not required to sum to 1, but are normalised internally.

Error Modelling

Sim3C models three forms of experimental noise: machine-based sequencing error, the formation of spurious ligation products and the contamination of PL libraries with WGS read-pairs.

To simulate machine-based sequencing error, the paired-end mode from art_illumina [15] has been reimplemented as a Python module (`Art.py`). This approach was taken as delegating read-pair generation to native invocations of art_illumina proved cumbersome. More explicitly, a loosely coupled solution (via subprocess calls but without an IPC mechanism) lacked sufficient control to generate PL read-pairs in an efficient and robust manner. On the other hand, tightly coupling Sim3C to the ART C/C++ source code (i.e. implementing hooks) would have left Sim3C vulnerable to changes in a non-public external API (i.e. a codebase without formal definition or guarantee of stability). Reimplementation also meant Art's many empirically derived machine profiles are available for use by Sim3C, allowing equivalent treatment of machine-error when experiments involve both PL (Sim3C) and pure WGS (art_illumina) libraries.

The production of spurious ligation products is an inherent source of noise in PL library construction [29]. Sim3C models spurious pairs as the uniformly random ligation of any two cut-sites across all source genomes. While this process disregards cellular organisation, it respects the relative abundance of chromosomes. Spurious pairs, and to a lesser extent sequencing error, represent an important confounding signal to downstream analyses that attempt to infer the cellular or chromosomal organisation of DNA sequences.

Lastly, conventional WGS read-pairs represent a source of contamination within a PL library, which even after Hi-C enrichment steps, are not completely eliminated. The rates at which spurious and WGS read-pairs are injected into a simulation run are controllable by the end-user.

Simulation modes

Since Hi-C was first introduced [22], the development of variants and extensions has been continual [12, 27, 34, 35]. Variants have often strived to further enhance the discriminatory power of the original experiment, while seemingly adding yet more complexity to an already challenging protocol (*in-situ* DNase Hi-C, sciHi-C) [35]. Others instead have sought compromise, with the aim of lessening the burden on the laboratory (Meta3C). While not considering more recent and complex extensions, Sim3C offers three simulation modes: traditional Hi-C, Meta3C and DNase Hi-C. The first two of these modes were chosen as representing the fundamental basis (traditional Hi-C) and an attractive and pragmatic simplification of the original (Meta3C). The third mode (DNase Hi-C) replaces the restriction endonuclease driven production of the free-ends, used to form PL products, with an ideally-free process of DNA fragmentation. In the laboratory, this ideally-free process could be carried out by DNase digestion or mechanical shearing via sonication.

The most notable difference between the methods of Hi-C and the more recent Meta3C, is that after restriction digest, Hi-C employs additional steps leading to the incorporation of biotin tags at each PL junction. This biotinylation permits Hi-C libraries to be subsequently enriched for fragments containing PL junctions by streptavidin-mediated affinity purification. Without enrichment, the simpler Meta3C protocol results in a gross mixture of both WGS and PL read-pairs, where only a small percentage of the total read-pair yield (approx. 1%) will possess PL junctions [23]. The enrichment process within Hi-C, however, is not perfectly efficient and WGS read-pairs are still observed (approx. 10–50% of reads contain a PL product) [23]. DNase Hi-C replaces restriction digest with a non-specific endonuclease (e.g. DNase I) [24] or mechanical DNA shearing process (e.g. sonication) [12]. In this operational mode, Sim3C treats DNA cleavage as a completely unbiased (free) process and as such all genomic positions have equal probability of participating in proximity ligation events.

Within Sim3C, each of the three methodological variations is conceptualised as a sequencing strategy (figure 1) and each iteration of a strategy produces one read-pair (PL or WGS in origin). For all strategies, an iteration begins by drawing a 3-tuple of insert parameters: length, direction and junction point (L_{ins}, dir, x_{junc}).

After obtaining insert parameters, the Hi-C strategy (figure 1a) first tests if the insert will represent a WGS or PL read-pair ($\sim Bern(p_{eff})$), where efficiency p_{eff} is defined in the sense of enrichment. When $p_{eff} = 1$, there is perfect filtering and all WGS read-pairs are eliminated from the experiment. In the case of WGS, the iteration reaches an end-point and the simulation emits a conventional read-pair drawn from the community definition. In the case of PL, a cut-site 3-tuple is drawn (gen_1, chr_1, x_1), where the categorical distribution over chromosomes is weighted by relative abundances (A) and chromosomal copy-numbers (n_{cpy}); genomic position is sampled uniformly from the set of restriction sites $(sites(chr_1))$; and parent genome (gen_1) is implicit from the chromosome. Next, a spurious ligation test is performed ($\sim Bern(p_{spur})$). If a spurious event has occurred, the 3-tuple defining the second cut-site (gen_2, chr_2, x_2) is drawn i.i.d. as the first. If not spurious, next a test for inter-chromosomal (*trans*) ligation is performed. Only source chromosome and position (chr_2, x_2) need be drawn as the second genome is implicitly the same as the first $(gen_2 = gen_1)$. Here, chr_2 is selected without replacement from the set of chromosomes of genome (gen_1), where the categorical distribution is adjusted by removal of chr_1. Finally, an intra-chromosomal (*cis*) ligation must have occurred. As now both genome and chromosome are implicit $(gen_2 = gen_1, chr_2 = chr_1)$, all that is left is to draw genomic position x_2. The pair of positions (x_1, x_2) are constrained by their separation $(s = |x_2 - x_1|)$, which is represented by a mixture model of the geometric and uniform distributions (equation 1). This relation possesses rapid falloff with increasing separation and non-zero probability

for all chromosomal positions, as has been commonly observed in real experimental data [10, 22].

$$Pr(X = s|\alpha, \beta, l) = \beta(1-\alpha)^s\alpha + (1-\beta)/l \quad (1)$$

where β is a mixing parameter, α the geometric distribution shape parameter and l chromosome length.

For Meta3C (figure 1b) after insert parameters are determined, in the same fashion as a regular WGS read, an initial free genomic position is drawn (chr_1, x_1^*), uniformly distributed over the extent of chr_1 rather than only over its cut-sites. In real datasets, it has been observed that neither the restriction digestion nor the re-ligation of free ends are perfectly efficient. Taken as independent probabilities, in our model we conceptualise their joint occurrence as an efficiency factor, p_{eff} and a Bernoulli trial $(Bern(p_{eff}))$ determines whether a sequence read is successful in containing an observable proximity ligation event. Failing this coverage test relegates the iteration and end-point and emit a WGS read-pair. Successful candidates instead continue akin to the Hi-C decision tree, beginning with the test for spurious ligation.

For both Hi-C and Meta3C, PL read-pairs are produced by joining the free-ends drawn above as defined by the fragment parameters (figure 2a). Here the location of the PL junction within the insert is determined by x_{junc}. At the junction, Hi-C differs from Meta3C as the process of biotinylation results in the duplication of the restriction cut-site overhang sequence. The overhang duplication in Hi-C is included in the simulation.

DNase Hi-C is handled similarly to traditional Hi-C, with the exception that, as *in-silico* digestion trivially leads to all sites, the simulated digestion is unnecessary to perform and positions can be drawn directly from the uniform distribution over the interval $[0..L_{chr})$. Site duplication, attributable to the likely production of random overhangs in this scenario, is not presently simulated.

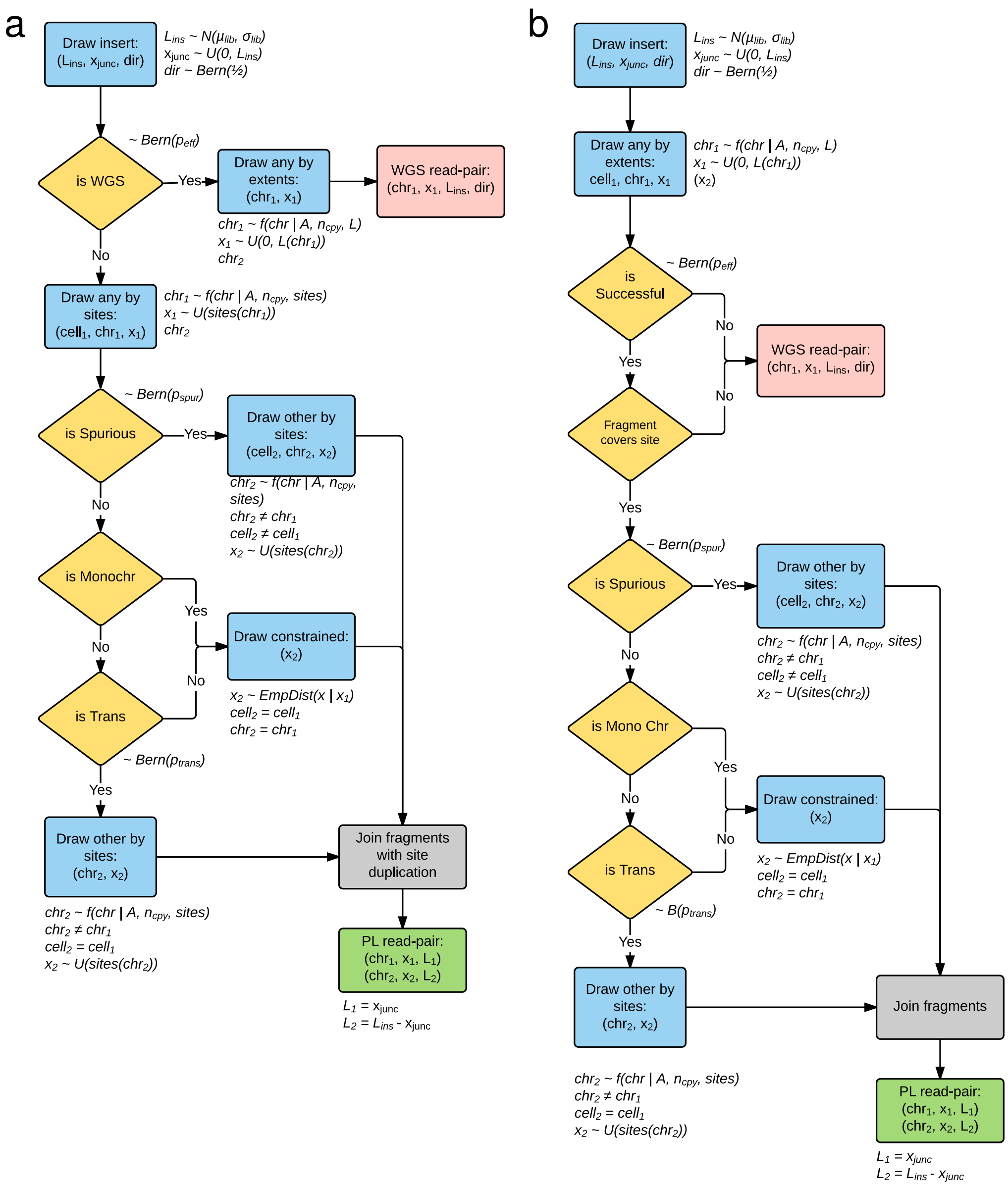

Figure 1. Logical schema used within Sim3C. (**a**) Hi-C and (**b**) Meta3C simulation strategies. Gold diamonds represent simple Bernoulli trials. Blue boxes represent sampling distributions defined by runtime input data (community profile, genomic sequences, enzyme) and the empirically derived distribution for intra-chromosome (*cis*) interaction probability (equation 1). Logical end-points to a single iteration of either algorithm are represented as red (producing a WGS read-pair) and green boxes (producing a PL read-pair). Due to the elimination of the biotinylation step, Meta3C does not produce a duplication of the restriction cut-site overhang (grey boxes).

Structurally related interactions

Independent of any 3D structure that might exist, the primary and most frequently observed interactions are those which occur along a chromosome (intra-arm) (figure 2b), seen as the primary ($y \simeq x$) diagonal in the contact map. Sim3C can approximate the

less frequent interactions occurring between chromosomal arms (inter-arm) [19], which are visible as anti-diagonal ($y \simeq L - x$) in the contact map.

At progressively smaller scales, the hierarchical 3D folding of DNA into topologically associated domains (TADs) produces overlapping regions of interaction visible in the contact map as block-like intensity modulations. Though the agents responsible for their formation vary [1,3], the characteristic patterns evident in real-data derived 3C contact maps have been observed across all three domains [10,19,40]. Sim3C can optionally approximate the sense of TAD related modulation by means of a recursive stochastic process.

Our approximation of hierarchical folding begins from the full extent L of a chromosome (figure 2c). Folding is portrayed by the division of the interval $[0..L)$ into a set of non-overlapping sub-intervals $\{[0, x_1), [x_1, x_2), \cdots, [x_{n-1}, x_n)\}$, the number and widths of which are drawn at random $(U(l_{min}, l_{max}), U(n_{min}, n_{max}))$. The procedure is then recursively applied to each sub-interval until a depth d, producing a nested set of coverings of the full interval $[0..L)$ at progressively finer scales. Across this hierarchical collection each interval is assigned a uniformly distributed random probability p_i and empirical distribution $f_i(s|\theta_i)$ (equation 1) for separation s parameterised by shape parameter α_{TAD} and interval length $l_{inv} = x_{i+1} - x_i$, where $\theta = (\alpha_{TAD}, \beta, l_{inv})$.

The process of drawing samples of separation begins by determining the set of intervals $\{l_{inv}\}$ which contain an initial point x_0. The intervals, as tuples $(p_i, f_i(s|\theta_i))$, then form a categorical distribution (equation (7)), from which a governing distribution $f_i(s|\theta_i)$ is drawn and finally a sample of separation is taken, $s \sim f_i(s|\theta_i)$. To efficiently sample from the full collection, an interval-tree data structure is employed. When queried, an interval-tree returns the set of intervals $\{l\}$ overlapping a position x in order $O(\log n + m)$, where n is number of intervals and m is number of intervals returned by the query.

$$\mathbf{f} = \{f_0(s|\theta_0), f_1(s|\theta_1), \cdots, f_i(s|\theta_i)\} \tag{2}$$

$$N = \text{number of distributions} = |\mathbf{f}| \tag{3}$$

$$\mathbf{p} = \{p_0, p_1, \cdots, p_i\} \tag{4}$$

$$p_i \sim U(0,1) \text{ and } \sum p_i = 1 \tag{5}$$

$$n \sim Cat(N, \mathbf{p}) \tag{6}$$

$$f(s|n) = \prod_{i=0}^{N-1} f_i(s|\theta_i)^{[i=n]} \tag{7}$$

where $[i = n]$ is the Iverson bracket.

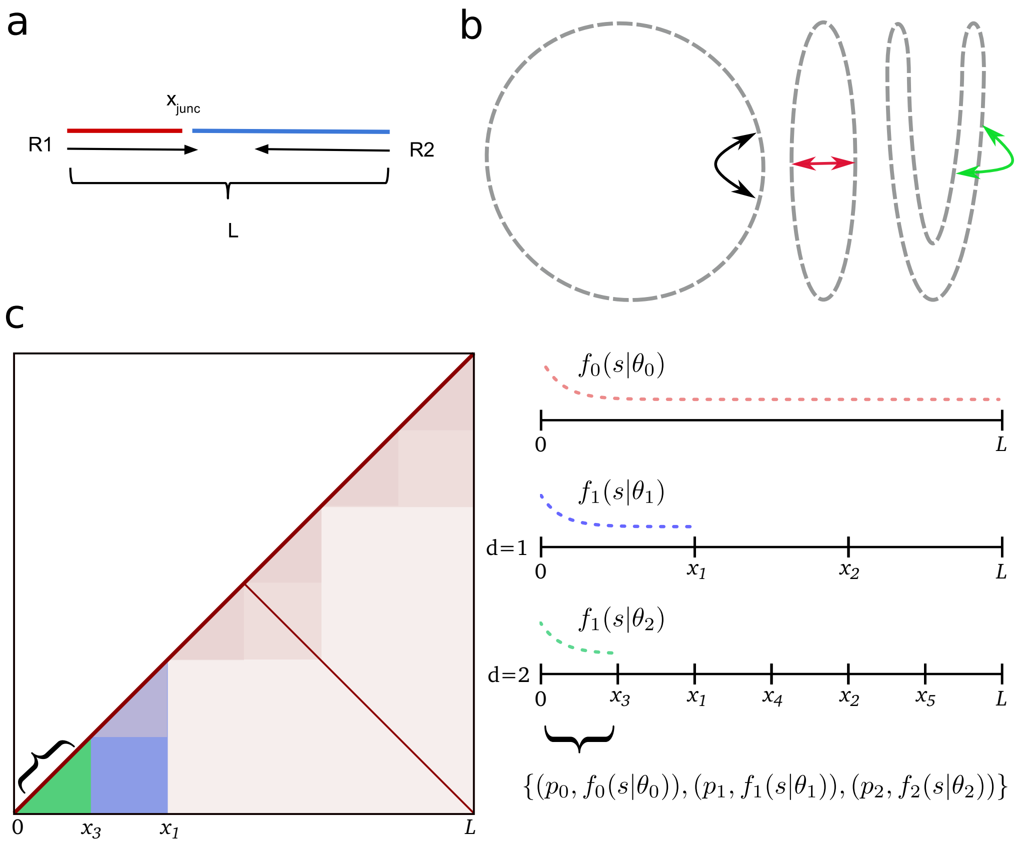

Figure 2. Model details. Generation of proximity ligation inserts (**a**) involves joining two randomly drawn parts (red and blue), from which the read-pair (R1, R2) is then simulated. The junction point (x_{junc}) varies over the interval $[0..L)$ and reproduction of read-through events is possible. For an unbounded chromosome (**b**) (circular here), besides strictly primary separation (black arrow) spatial proximity can be induced from successive folding (red, green arrows). When the spatial arrangement is consistent across the population of cells, this will be observable as modulations in the contact frequencies. Sim3C models simple structurally related modulation of observed contact frequencies (**c**). Beyond primary interactions forming the main diagonal, users can reproduce inter-arm mediated anti-diagonals. Finer scale modulations attributed to topologically associated domains (TADs) can optionally be randomly simulated. Primary interactions $f_0(s|\theta_0)$ (equation 1) cover the full interval $[0, L)$. Each level of recursion ($d = 1, 2 \cdots n$) generates a finer set of intervals, to which a distribution $f_i(s|\theta_i)$ and probability p_i is assigned. The final covering of intervals each define a range (green, curly braces) over which a set of probabilities and empirical distribution pairs govern interaction separation s.

Example scenarios

In the following, three use-cases are presented to demonstrate aspects of the resulting simulation output: bacterial genome, multi-chromosomal eukaryotic (yeast) genome, and metagenome. For each use-case, 3C contact maps have been used to pit simulation output against the corresponding real experimental data (table 1).

Bacterial

A monochromosomal bacterial genome is perhaps the simplest scenario to which proximity ligation methods have been applied, making for a sensible entry point from which to make comparison. Due to the smaller extent, a bright and high resolution contact map (10 kbp bin size) is possible for a practical volume of sequencing data, potentially revealing fine detail not easily discerned with larger bin sizes (50-100 kbp bin size).

The genome of *Caulobacter crescentus* NA1000, a model organism in the study of cellular differentiation and regulation of the cell cycle, is comprised of a single 4 Mbp circular chromosome [28]. Deep Hi-C sequencing of *C. crescentus* has been used to explore the degree to which bacterial chromosomes can be regarded as organised and provided evidence for the existence of so called chromosomal interaction domains (CIDs) [19]. As a prokaryotic analog of topologically associated domains (TADs) from eukaryotic literature [1, 30, 33], these regions are believed to promote intra-domain loci interactions and thereby act to functionally compartmentalize the genome. This chromosomal structure was observed to be at once disruptable through rifampicin mediated inhibition of transcription and malleable by the movement of highly expressed genes [19].

For the raw contact map of *C. crescentus*, prominent rectilinear features are apparent for both real and simulated traditional Hi-C sequencing data (figure 3a,b), while notably for simulated unrestricted Hi-C the field is much smoother (figure 3c). Within the Sim3C model, a single distribution governs both intra- and inter-arm interactions. Inspection of the real-data contact map (figure 3a) suggests that the true relationship governing inter-arm interactions is more dispersed. This perhaps is not surprising, where different arms associating spatially possess a greater number of potential configurations than can be taken on by the primary chromosome backbone. Additionally for the real contact map, long-range interactions away from either diagonal can be seen to drop to a lower threshold than that produced from simulation.

Within the unrestricted Hi-C map, the fine zero-intensity rectilinear features are a direct result of poor mappability (non-unique sequence), where their small size reflects the extent of the non-unique regions (example: rRNA genes) and the single base-pair resolution of the less constrained read generation process. The process of enzymatic digestion is the only difference between the unrestricted and traditional Hi-C simulation models. The clear contrast in their contact maps is thus a combination of factors either directly inherent to digestion (cut-site density) or a byproduct of downstream bioinformatics analysis (e.g. filtering heuristics). Though the problem of mappability exists for any reference based representation, for real and simulated traditional Hi-C, zero-intensity rectilinear features mark regions devoid of cut-sites over at least 10 kbp.

Enabling TAD approximation in simulated traditional Hi-C (figure 3d) has the effect of modulating map intensity in a manner not particularly distinct from that produced purely from experimental/workflow bias. Discriminating between these two feature sources; one representing experimental signal, the other representing noise; demands attention when developing solutions to problems such as normalisation. Contact map normalisation methods, whether based upon explicit or implicit bias models [38], may leave behind remnants of noise-related features from either a lack of convergence or model limitations. Downstream inferencing should therefore not be made under an assumption of bias-free signal.

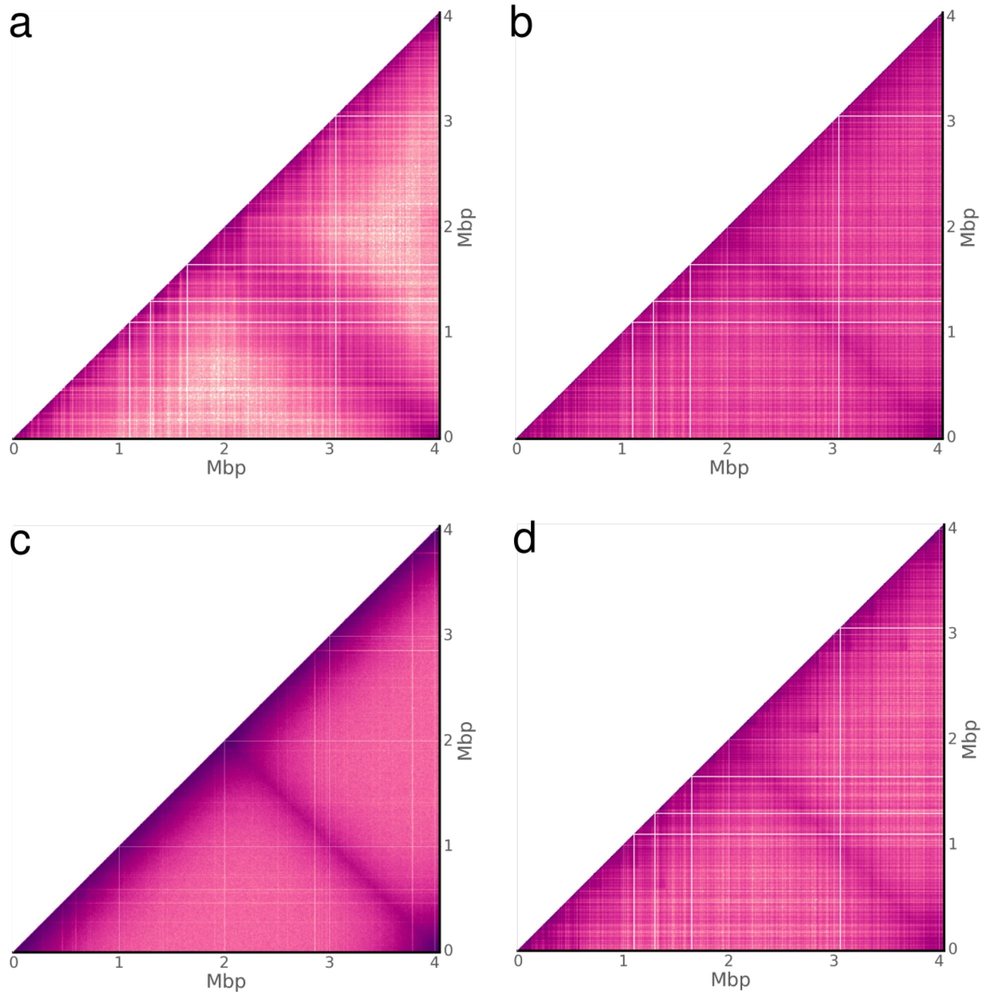

Figure 3. Bacterial contact maps. Observed Hi-C interactions for the monochromosomal genome of *Caulobacter crescentus* NA1000. Comparing (**a**) real experimental data [19], to the three simulation choices (**b**) traditional Hi-C, (**c**) DNase Hi-C and (**d**) traditional Hi-C with TADs enabled. Sharp rectilinear modulations of the intensity within (**a**) and (**b**) indicate a reduction in PL observations within a given bin. Not due to 3D chromosome structure, rather such features can be attributed largely to mappability and low cut-site density. (**c**) Without an enzymatic constraint a significantly smoother field is apparent, yet still susceptible to mappability. (**d**) Enabling topologically associated domains (TADs) highlights the similarity between features produced merely from biases and what could be truly associated with 3D structure.

Eukaryotic

The eight chromosomes of the 15.4 Mbp genome of the native xylose-fermenting yeast *Scheffersomyces stipitis* CBS 6054 [16] range in size from 970 kbp to 3.5 Mbp. The organism was one of 16 yeasts included in a synthetic community to explore the application of Hi-C sequencing to deconvolving metagenomic assemblies [6] and is divergent enough from other synthetic community members to permit unambiguous read mapping, and thus act as a proxy for a clonal experiment.

From the contact map of real Hi-C data (figure 4a), it can be seen that the rates of intra-chromosomal and inter-chromosomal interactions are roughly equivalent in magnitude. Across the eight chromosomes of *S. stipitis*, there is significant uniformity in the degree of physical intimacy within and between all chromosomes. The subtleties of this chromosomal organisation reveals a self-similar "fuzzy-x" pattern repeated between all chromosomes across the contact map. The convergence point within the pattern is attributed to centromere-SPB binding and has been used to predict centromere locations [42]. It has been shown that the physical constraints generated from the interaction of centromeres to the spindle pole body (SPB) and telomeres to the nuclear envelope are sufficient to explain a number of experimental observations in real data [13, 43]. As Sim3C was derived from study of bacterial datasets, our simulation model does not currently include a notion of these higher organism physical constraints. Consequently, the contact map derived from simulated traditional Hi-C sequencing elicits a flat field (figure 4b), where the intensity variation that does exist is a byproduct of aforementioned factors such as mappability and cut-site density. For the runtime parameters employed, the rate of intra-chromosomal contact is higher than that of inter-chromosomal, making clear the boundaries between the eight chromosomes (figure 4b). Though our model is presently incomplete for higher organisms, there remains a potential utility as an analytical or simply observational prior.

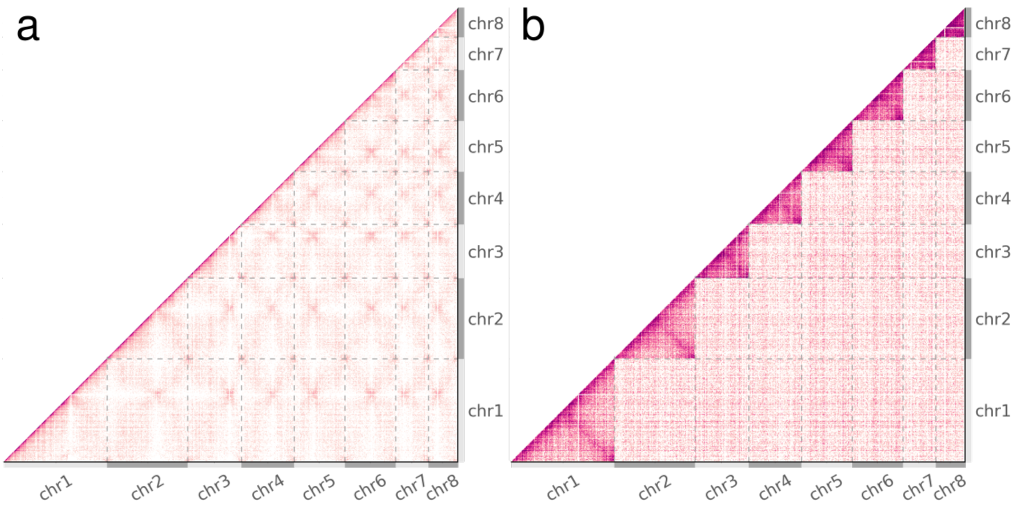

Figure 4. Eukaryotic contact maps. Observed Hi-C interactions (**a**) real and (**b**) simulated data from the eight chromosome genome of the budding yeast *Scheffersomyces stipitis* CBS 6054 [6]. Grey dashed lines and alternating light and dark grey axes demarcate the boundaries between chromosomes. (**b**) Simulated data elicits a flat field and the clearly evident higher rate of intra- to inter- interactions makes for easily observable chromosomal boundaries within the map. (**a**) Contrastingly for real data, the similar rates of intra-chr and inter-chr interactions reveals the physical constraints imposed by centromere-SPB tethering on all eight chromosomes [42].

Metagenomic

In the deconvolution of metagenomes, proximity ligation methods hold great potential as new sources of information and have been investigated by the construction and sequencing of synthetic communities [4, 6, 27]. We selected two previously constructed synthetic bacterial communities, one employing traditional Hi-C and the other Meta3C

(table 1). Intended as “proof of concept” experiments, neither community reflects a real environment, but rather were intended to be easily interpreted and include interesting features, such as: range of GC, single and multi- chromosomal genomes and strain-level divergence. The Hi-C community involved five genotypes from four species, one genome of two chromosomes (*B. thailandensis*), *E. coli* strains BL21 and K12 (Average Nucleotide Identity, ANI 99%) and a wide overall GC range of 37-68% (table 2). Of lower complexity, the Meta3C community involved three genomes from three species, included one genome of two chromosomes (*V. cholerae*) and had a narrower GC range of 44-51% (table 3). Relative to the single genome experiments above, a lower depth of sequencing resulted in a lower overall contact map intensity (figure 5). This is particularly the case for Meta3C, where, by the nature of the method, a large proportion (approx. 99%) of the sequencing yield is in reality conventional WGS read-pair data [27]. As a direct result, in binning the Meta3C dataset, there were insufficient counts to fully establish finer detail within the contact maps, leaving a smoother appearance.

As with single-genome experiments, metagenomic contact maps are locally modulated by factors such as mappability and cut-site density. Importantly now for metagenomes, the factors of relative abundance and GC content interact to alter the observed intensity of each chromosome within the contact map.

As a first approximation and assuming agreement in nucleotide sampling frequency, we expect $n_0 = L/4^\lambda$ recognition sites for an enzyme of site length λ and DNA sequence length L. The degree to which an enzyme and DNA sequence deviate from this estimate could be described as how well they match, $m = n_x/n_0$. Poorer quality matches ($m < 1$) occur when an enzyme's recognition site is underrepresented, while conversely, better quality matches ($m > 1$) describe a situation of more recognition sites than expected.

When multiple chromosomes are taken as a community, the relative proportion of sites from each represents an observational bias when conducting 3C-based experiments. For community C, the number of sites n_x from chromosome x determines the number of potential PL pairings N_x within C which involve x (equation 8). The number of intra-chromosomal and inter-chromosomal potential pairs thus respectively vary quadratically and linearly with n_x. Regarding the process of observing a PL event (read-pair) from the community as a random draw with replacement, and the selection pool as comprised of all potential events from all chromosomes, then variation in match quality constitutes a per-chromosome bias. In real laboratory experiments, the composition of the selection pool is further modified by variation in other factors, such as cellular lysis efficiency, unintended DNA fragmentation and relative abundance. In particular, when relative abundances A are introduced, the odds of observing a PL event involving chromosome x is then proportional the product $p_x \propto A_x N_x / N_C$. Although the processes of intra-chromosomal, inter-chromosomal, and inter-cellular (spurious) ligation are treated independently in our simulation model, in this manner, per-chromosome intensity (observation rate of chromosome x) can vary significantly within a metagenome.

$$N_x = n_x^2 + n_x \sum_{n_y \in C \setminus n_x} n_y \tag{8}$$

Though the original laboratory experiments reported by Beitel et al. 2014 and Marbouty et al. 2014 intended o create synthetic communities with uniform relative abundances, in practice each possesses a non-uniform profile. The variation in GC content is largest for the Hi-C experiment and together with non-uniform relative abundances produces a wide range of chromosome intensity for both real and simulated data (figure 5a,b). For both the real and simulated Hi-C maps, the frequent observation of PL events involving *P. pentosaceus* (Pp) and *L. brevis* (Lb), suggests the possibility

that inter-cellular interaction is significant. Within the simulated map at least, inter-cellular pairs are produced exclusively through the process of spurious ligation (noise) and are observed at a higher rate than in the real data, indicating that as expected, spurious ligation rates across species are correlated with their relative abundances.

Further for the Hi-C data, the two-chromosome genome of *B. thailandensis* (Bt1, Bt2) (figure 5a) has a greater rate of inter-chromosomal interaction than expected from comparing it to simulation (figure 5b). Meanwhile, the clear delineation of *E. coli* strains BL21 and K12 ($ANI > 99\%$), with little inter-cellular signal, helps to support the notion that the inter-chromosomal interactions observed between *B. thailandensis* chromosomes ($ANI \simeq 83\%$) are real and not a by-product of inadequate filtering.

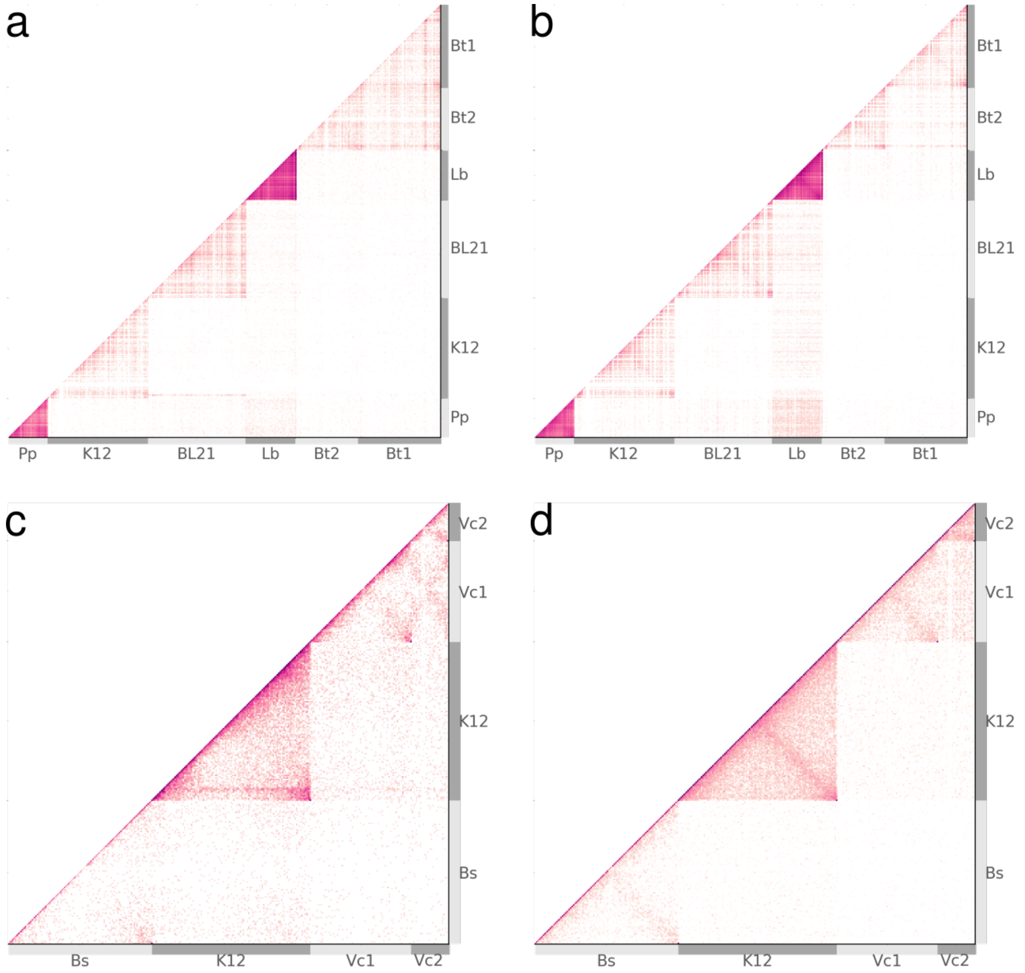

Figure 5. Metagenomic contact maps. From synthetic microbial communities, raw contact maps from real (**a**) and simulated (**b**) traditional HiC, and real (**c**) and simulated (**d**) Meta3C. Chromosome boundaries are demarcated by alternating light and dark grey bands (tables 2, 3), while the small plasmids of *L. brevis* are omitted for clarity. Although the original works [4, 27] intended uniform abundance, the results exhibit significant variation in abundance. Lysis efficiency (not modelled) and enzyme suitability are significant factors contributing to the overall intensity of a given chromosome. For more abundant members of the Hi-C community (*P. pentosaceus* and *L. brevis*), signal due only to spurious ligation can appear to suggest inter-cellular interactions when none are present (**b**).

Limitations and future work

Sim3C in its current form has several limitations, some of which present opportunities for future work. Sim3C's repertoire of structural features is currently limited to those found in microbes - circular and linear chromosomes with randomly generated approximations of self-associating domains (CIDs/TADs). Sim3C does not model structural features observed in larger, more complex genomes (CTCF/cohesin loops, A/B compartments, chromosome territories) [22, 36]. Such features are becoming increasingly well characterised [41] and a simulator capable of modelling these features would surely be valuable. Mammalian genomes are much larger than microbial genomes

Authors	Type	Method	Accession	Sequencing details	Mapped reads
Beitel et al [4]	Synthetic bacterial metagenome	Hi-C	SRX377733	MiSeq 160bp PE<br>insert range: 280-420bp<br>enzyme: HindIII	20552775
Burton et al [6]	Synthetic yeast metagenome	Hi-C	SRX527868	HiSeq2500 100bp PE<br>insert range: 450-550bp<br>enzyme: HindIII	9704944
Le et al [19]	Single bacterial genome	Hi-C	SRX263925	HiSeq2000 40bp PE<br>insert range: 200-600bp<br>enzyme: NcoI	22324360
Marbouty et al [26]	Synthetic bacterial metagenome	Meta3C	doi:10.5061/dryad.gv595	HiSeq2000 100bp PE<br>insert range: 400-800bp<br>enzyme: HpaII	7975740

Table 1. Real Hi-C and Meta3C data-sets used within this work. The total off-diagonal weight of the contact map was used to calibrate the amount of simulated sequencing required to approximately match the outcome of the real experiments.

however, and additional work to improve scalability of Sim3C will likely be required.

Some features of microbial eukaryotes, such as the point centromeres found in budding yeast genomes [7] are computationally simpler [13, 42] yet remain unmodelled in Sim3C. The addition of these sorts of model details would be best supported by introducing model initialisation via external data (experimental observations, motif detection, cell phase), which subsequently would require extension of the community profile definition. Careful design would be required to ensure these features could be added without compromising ease-of-use.

Methods

Reference Data

To compare Sim3C against real experiments, we obtained previously published experimental read-pair datasets (table 1) and their accompanying reference genomes (tables 2, 3) from public archives. In the case of the single genome project of *Caulobacter crescentus* CB15 [19], sequencing data derived from untreated swarmer cells was chosen and the laboratory strain *C. crescentus* NA1000 (acc: NC_011916) was used as the reference genome. For the yeast genome, the completed eight chromosome genome of *Scheffersomyces stipitis* CBS 6054 was used as a reference (acc: PRJNA18881) and the respective reads were extracted from the MY16 yeast synthetic metagenome [6] by direct mapping with BWA MEM. Extraction by mapping in isolation was employed as *S. stipitis* was the second furthest phylogenetically removed yeast in the synthetic community and was the most contiguous (N50: 60kbp) from the whole synthetic community de novo metagenomic WGS assembly.

Read Generation

Experimental parameters used in read simulation were set to agree as closely as reasonably possible to the respective real experiments, employing the same read length and restriction enzyme (table 1). In each experiment, the published fragment size range was approximated by a normal distribution (table 4). For ease of reproducibility, a

Name	Replicons	Accession	Chr abbr.	A	n_{cpy}	%GC	n_x	m
Burkholderia thailandensis E264	2	NC_007651<br>NC_007650	Bt1<br>Bt2	0.054	1	67.29<br>68.07	225<br>144	0.24<br>0.20
Escherichia coli BL21	1	NC_012892	BL21	0.242	1	50.83	508	0.46
Escherichia coli K12 DH10B	1	NC_010473	K12	0.166	1	50.78	568	0.50
Lactobacillus brevis ATCC 367	3	NC_008497<br>NC_008498<br>NC_008499	Lb<br>-<br>-	0.436	1	46.22<br>38.64<br>38.51	629<br>3<br>16	1.12<br>0.92<br>1.84
Pediococcus pentosaceus ATCC 25745	1	NC_008525	Pp	0.102	1	37.36	863	1.93

Table 2. Synthetic Hi-C community. A synthetic community used to demonstrate the utility of Hi-C sequencing data in resolving a microbial metagenome [4]. It is composed of 5 bacteria, including two closely related strains (*E. coli* K12 and BL21), a genome with two plasmids (*L. brevis*) and a two-chromosome genome (*B. thailandensis*). A is relative abundance, n_{cpy} is copy number, n_x is number of restriction sites, and $m = n_x/n_0$ is match quality between chromosome and enzyme choice: $m < 1$ is worse, $m > 1$ is better.

Name	Replicons	Accession	Chr abbr.	A	n_{cpy}	%GC	n_x	m
Bacillus subtilis subsp. subtilis str. 168	1	NC_000964	Bs	0.123	1	43.51	14529	0.88
Escherichia coli str. K-12 substr. MG1655	1	NC_000913	K12	0.562	1	50.79	24311	1.34
Vibrio cholerae O1 biovar El Tor str. N16961	2	NC_002505<br>NC_002506	Vc1<br>Vc2	0.332	1	47.70<br>46.91	5909<br>1802	0.51<br>0.43

Table 3. Synthetic Meta3C community. A synthetic community used to demonstrate the utility of Meta3C sequencing data in resolving a microbial metagenome [26,27]. It is composed of three bacteria with one possessing two chromosomes. A is relative abundance, n_{cpy} is copy number, n_x is number of restriction sites, and $m = n_x/n_0$ is match quality between chromosome and enzyme choice: $m < 1$ is worse, $m > 1$ is better.

Experiment	Insert μ (bp)	Insert σ (bp)	Anti rate	Spurious rate	Trans rate	Reads ($\times 10^6$)
Beitel et al	300	50	0.2	0.05	0.1	7
Burton et al	400	50	0.2	0.5	0.15	1.5
Le et al	400	100	0.2	0.2	0.1	22
Marbouty et al	600	100	0.2	0.2	0.2	7.5

Table 4. Runtime simulation. Parameters supplied to Sim3C during read generation.

single random seed (1234) was used in all simulations. As our intent was primarily to demonstrate functionality, rates of inter-chromosomal and spurious events were adjusted per-experiment only through a qualitative process. For simulation of metagenomic datasets, relative abundances were estimated by mapping real experimental reads to the respective reference genomes. From each real experiment, the off-diagonal weight of the resulting contact map was used to calibrate the amount of simulated sequencing required to achieve roughly equivalent intensity (table 4). Both real and simulated read-pair datasets were mapped to their respective reference genomes using BWA MEM (v0.7.15-r1140, RRID:SCR_010910) [21]

Contact Maps

Contact maps were produced using our own tool (`contact_map.py`), where heatmap intensity was plotted as log-scaled observational frequency. All aligned reads were subject to the same basic filtering criteria: BWA MEM mapq > 5 and alignment length $\geq 50\%$ of read length, with the added restriction that read alignments must have begun with a match. For methods which employed a restriction enzyme (traditional Hi-C, Meta3C), we constrained the maximum allowable distance from an aligned read to the nearest upstream cut-site. Calculated per chromosome, this distance constraint could not exceed two-fold the median cut-site spacing. Rather than simply delete the primary diagonal for the sake of reducing the displayed dynamic range in figures, we instead to reduced its intensity by categorizing properly paired reads with an estimated fragment size of less than 2 of the reported mean as being conventional WGS (non-PL) reads and ignored them. The resolution of contact maps was adjusted between experiments so as to present a sufficiently bright image without undue loss of resolution. The contact map bin sizes employed were: 10000 bp for the single bacterial genome, 25000 bp for the yeast genome and 40000 bp for the Hi-C and Meta3C metagenomes (tables 2, 3).

Availability of data and materials

Snapshots of the supporting code are available from the GigaScience repository, GigaDB [32].

Availability of supporting source code and requirements

- Project name: Sim3C
- Release version: 0.1
- Project homepage: https://github.com/cerebis/sim3C
- RRID: SCR_015772
- DOI: 10.5281/zenodo.1030812
- Operating system: Platform independent
- Programming languages: Python 2.7
- License: GNU GPL v3

Declarations

List of abbreviations

- IPC - interprocess communication
- PL - proximity ligation
- WGS - whole genome shotgun
- CID - chromosomal interaction domain
- TAD - topologically associated domain
- $Bern(x)$ - Bernoulli distribution
- $U(x)$ - uniform distribution
- $N(\mu, \sigma)$ - normal distribution
- *cis* - intra-chromosomal
- *trans* - inter-chromosomal

Ethics approval and consent to participate

Not applicable

Competing interests

The authors declare that they have no competing interests.

Funding

This work was supported under Australian Research Council's Discovery Projects funding scheme (project number: LP150100912, CI: Djordjevic, SP). The NeCTAR Research Cloud is an Australian Government project conducted as part of the Super Science initiative and financed by the and the Education Investment Fund (EIF) and National Collaborative Research Infrastructure Strategy (NCRIS).

- https://www.education.gov.au/education-investment-fund
- https://www.education.gov.au/national-collaborative-research-infrastructure-strategy-ncris

Authors contributions

MD designed and implemented Sim3C and wrote the manuscript and prepared figures. AD assisted in the design and contributed to the manuscript.

Acknowledgements

We thank Steven P. Djordjevic for his support and helpful discussions. This work was supported by the AusGEM initiative, a collaboration between the NSW Department of Primary Industries and the ithree institute. We acknowledge the use of computing resources from the NeCTAR Research Cloud, the QCIF and the UTS eResearch Group.

- http://www.nectar.org.au
- http://www.qcif.edu.au
- https://eresearch.uts.edu.au

References

1. R. D. Acemel, I. Maeso, and J. L. Gómez-Skarmeta. Topologically associated domains: a successful scaffold for the evolution of gene regulation in animals. *Wiley Interdiscip. Rev. Dev. Biol.*, 2 Mar. 2017.

2. F. E. Angly, D. Willner, F. Rohwer, P. Hugenholtz, and G. W. Tyson. Grinder: a versatile amplicon and shotgun sequence simulator. *Nucleic Acids Res.*, 40(12):e94–e94, 1 July 2012.

3. A. Badrinarayanan, T. B. K. Le, and M. T. Laub. Bacterial chromosome organization and segregation. *Annu. Rev. Cell Dev. Biol.*, 31(1):171–199, 1 Jan. 2015.

4. C. W. Beitel, J. M. Lang, I. F. Korf, R. W. Michelmore, J. A. Eisen, and A. E. Darling. Strain- and plasmid-level deconvolution of a synthetic metagenome by sequencing proximity ligation products. *PeerJ*, 2(12):e415, 1 Jan. 2014.

5. J. N. Burton, A. Adey, R. P. Patwardhan, R. Qiu, J. O. Kitzman, and J. Shendure. Chromosome-scale scaffolding of de novo genome assemblies based on chromatin interactions. *Nat. Biotechnol.*, 31(12):1119–1125, 3 Nov. 2013.

6. J. N. Burton, I. Liachko, M. J. Dunham, and J. Shendure. Species-Level deconvolution of metagenome assemblies with Hi-C-Based contact probability maps. *G3*, 4(7):1339–1346, 22 May 2014.

7. G. Cottarel, J. H. Shero, P. Hieter, and J. H. Hegemann. A 125-base-pair CEN6 DNA fragment is sufficient for complete meiotic and mitotic centromere functions in saccharomyces cerevisiae. *Mol. Cell. Biol.*, 9(8):3342–3349, Aug. 1989.

8. J. Dekker, K. Rippe, M. Dekker, and N. Kleckner. Capturing chromosome conformation. *Science*, 295(5558):1306–1311, 15 Feb. 2002.

9. M. Z. DeMaere and A. E. Darling. Deconvoluting simulated metagenomes: the performance of hard- and soft- clustering algorithms applied to metagenomic chromosome conformation capture (3c). *PeerJ*, 4(4):e2676, 1 Jan. 2016.

10. J. R. Dixon, S. Selvaraj, F. Yue, A. Kim, Y. Li, Y. Shen, M. Hu, J. S. Liu, and B. Ren. Topological domains in mammalian genomes identified by analysis of chromatin interactions. *Nature*, 485(7398):376–380, 11 Apr. 2012.

11. O. Dudchenko, S. S. Batra, A. D. Omer, S. K. Nyquist, M. Hoeger, N. C. Durand, M. S. Shamim, I. Machol, E. S. Lander, A. P. Aiden, and E. L. Aiden. De novo assembly of the aedes aegypti genome using Hi-C yields chromosome-length scaffolds. *Science*, 356(6333):92–95, 7 Apr. 2017.

12. M. Fullwood, P. Y. H. Huang, Y. Han, L. Handoko, S. Velkov, E. Wong, E. Cheung, X. Ruan, C.-L. Wei, M. J. Fullwood, and Y. Ruan. Protocol: Sonication-based circular chromosome conformation capture with next-generation sequencing analysis for the detection of chromatin interactions. *Protocol Exchange*, 14 Dec. 2010.

13. K. Gong, H. Tjong, X. J. Zhou, and F. Alber. Comparative 3D genome structure analysis of the fission and the budding yeast. *PLoS One*, 10(3):e0119672, 23 Mar. 2015.

14. X. Hu, J. Yuan, Y. Shi, J. Lu, B. Liu, Z. Li, Y. Chen, D. Mu, H. Zhang, N. Li, Z. Yue, F. Bai, H. Li, and W. Fan. pIRS: Profile-based illumina pair-end reads simulator. *Bioinformatics*, 28(11):1533–1535, 1 June 2012.

15. W. Huang, L. Li, J. R. Myers, and G. T. Marth. ART: a next-generation sequencing read simulator. *Bioinformatics*, 28(4):593–594, 15 Feb. 2012.

16. T. W. Jeffries, I. V. Grigoriev, J. Grimwood, J. M. Laplaza, A. Aerts, A. Salamov, J. Schmutz, E. Lindquist, P. Dehal, H. Shapiro, Y.-S. Jin, V. Passoth, and P. M. Richardson. Genome sequence of the lignocellulose-bioconverting and xylose-fermenting yeast pichia stipitis. *Nat. Biotechnol.*, 25(3):319–326, Mar. 2007.

17. B. Jia, L. Xuan, K. Cai, Z. Hu, L. Ma, and C. Wei. NeSSM: a next-generation sequencing simulator for metagenomics. *PLoS One*, 8(10):e75448, 1 Jan. 2013.

18. J. O. Korbel and C. Lee. Genome assembly and haplotyping with Hi-C. *Nat. Biotechnol.*, 31(12):1099–1101, Dec. 2013.

19. T. B. K. Le, M. V. Imakaev, L. A. Mirny, and M. T. Laub. High-resolution mapping of the spatial organization of a bacterial chromosome. *Science*, 342(6159):731–734, 8 Nov. 2013.

20. H. Li. lh3/wgsim. `https://github.com/lh3/wgsim`, 18 Oct. 2011. Accessed: 2017-3-21.

21. H. Li. Aligning sequence reads, clone sequences and assembly contigs with BWA-MEM. *arXiv.org*, q-bio.GN, 17 Mar. 2013.

22. E. Lieberman-Aiden, N. L. van Berkum, L. Williams, M. Imakaev, T. Ragoczy, A. Telling, I. Amit, B. R. Lajoie, P. J. Sabo, M. O. Dorschner, R. Sandstrom, B. Bernstein, M. A. Bender, M. Groudine, A. Gnirke, J. Stamatoyannopoulos, L. A. Mirny, E. S. Lander, and J. Dekker. Comprehensive mapping of long-range interactions reveals folding principles of the human genome. *Science*, 326(5950):289–293, 9 Oct. 2009.

23. M. Liu and A. Darling. Metagenomic chromosome conformation capture (3c): techniques, applications, and challenges. *F1000Res.*, 4(1377):1–9, 1 Jan. 2015.

24. W. Ma, F. Ay, C. Lee, G. Gulsoy, X. Deng, S. Cook, J. Hesson, C. Cavanaugh, C. B. Ware, A. Krumm, J. Shendure, C. A. Blau, C. M. Disteche, W. S. Noble, and Z. Duan. Fine-scale chromatin interaction maps reveal the cis-regulatory landscape of human lincRNA genes. *Nat. Methods*, 12(1):71–78, 1 Jan. 2015.

25. M. Marbouty, L. Baudry, A. Cournac, and R. Koszul. Scaffolding bacterial genomes and probing host-virus interactions in gut microbiome by proximity ligation (chromosome capture) assay. *Sci Adv*, 3(2):e1602105, Feb. 2017.

26. M. Marbouty, A. Cournac, J.-F. Flot, H. Marie-Nelly, J. Mozziconacci, and R. Koszul. Data from: Metagenomic chromosome conformation capture (meta3c) unveils the diversity of chromosome organization in microorganisms. 2014.

27. M. Marbouty, A. Cournac, J.-F. Flot, H. Marie-Nelly, J. Mozziconacci, and R. Koszul. Metagenomic chromosome conformation capture (meta3c) unveils the diversity of chromosome organization in microorganisms. *Elife*, 3(e03318):e03318, 17 Dec. 2014.

28. M. E. Marks, C. M. Castro-Rojas, C. Teiling, L. Du, V. Kapatral, T. L. Walunas, and S. Crosson. The genetic basis of laboratory adaptation in caulobacter crescentus. *J. Bacteriol.*, 192(14):3678–3688, July 2010.

29. T. Nagano, C. Várnai, S. Schoenfelder, B.-M. Javierre, S. W. Wingett, and P. Fraser. Comparison of Hi-C results using in-solution versus in-nucleus ligation. *Genome Biol.*, 16:175, 26 Aug. 2015.

30. E. P. Nora, B. R. Lajoie, E. G. Schulz, L. Giorgetti, I. Okamoto, N. Servant, T. Piolot, N. L. van Berkum, J. Meisig, J. Sedat, J. Gribnau, E. Barillot, N. Blüthgen, J. Dekker, and E. Heard. Spatial partitioning of the regulatory landscape of the x-inactivation centre. *Nature*, 485(7398):381–385, 17 May 2012.

31. Y. Ono, K. Asai, and M. Hamada. PBSIM: PacBio reads simulator–toward accurate genome assembly. *Bioinformatics*, 29(1):119–121, 1 Jan. 2013.

32. B. S. Pedersen, R. L. Collins, M. E. Talkowski, and A. R. Quinlan. Supporting software for "indexcov: fast coverage quality control for whole-genome sequencing", 2017.

33. B. D. Pope, T. Ryba, V. Dileep, F. Yue, W. Wu, O. Denas, D. L. Vera, Y. Wang, R. S. Hansen, T. K. Canfield, R. E. Thurman, Y. Cheng, G. Gulsoy, J. H. Dennis, M. P. Snyder, J. A. Stamatoyannopoulos, J. Taylor, R. C. Hardison, T. Kahveci, B. Ren, and D. M. Gilbert. Topologically associating domains are stable units of replication-timing regulation. *Nature*, 515(7527):402–405, 20 Nov. 2014.

34. V. Ramani, D. A. Cusanovich, R. J. Hause, W. Ma, R. Qiu, X. Deng, C. A. Blau, C. M. Disteche, W. S. Noble, J. Shendure, and Z. Duan. Mapping 3D genome architecture through in situ DNase Hi-C. *Nat. Protoc.*, 11(11):2104–2121, 1 Nov. 2016.

35. V. Ramani, X. Deng, R. Qiu, K. L. Gunderson, F. J. Steemers, C. M. Disteche, W. S. Noble, Z. Duan, and J. Shendure. Massively multiplex single-cell Hi-C. *Nat. Methods*, 14(3):263–266, 30 Jan. 2017.

36. S. S. P. Rao, M. H. Huntley, N. C. Durand, E. K. Stamenova, I. D. Bochkov, J. T. Robinson, A. L. Sanborn, I. Machol, A. D. Omer, E. S. Lander, and E. L. Aiden. A 3D map of the human genome at kilobase resolution reveals principles of chromatin looping. *Cell*, 159(7):1665–1680.

37. D. C. Richter, F. Ott, A. F. Auch, R. Schmid, and D. H. Huson. MetaSim: a sequencing simulator for genomics and metagenomics. *PLoS One*, 3(10):e3373, 8 Oct. 2008.

38. A. D. Schmitt, M. Hu, and B. Ren. Genome-wide mapping and analysis of chromosome architecture. *Nat. Rev. Mol. Cell Biol.*, 17(12):743–755, 1 Dec. 2016.

39. S. Selvaraj, J. R Dixon, V. Bansal, and B. Ren. Whole-genome haplotype reconstruction using proximity-ligation and shotgun sequencing. *Nat. Biotechnol.*, 31(12):1111–1118, Dec. 2013.

40. T. Sexton, E. Yaffe, E. Kenigsberg, F. Bantignies, B. Leblanc, M. Hoichman, H. Parrinello, A. Tanay, and G. Cavalli. Three-dimensional folding and functional organization principles of the drosophila genome. *Cell*, 148(3):458–472, 3 Feb. 2012.

41. T. J. Stevens, D. Lando, S. Basu, L. P. Atkinson, Y. Cao, S. F. Lee, M. Leeb, K. J. Wohlfahrt, W. Boucher, A. O'Shaughnessy-Kirwan, J. Cramard, A. J. Faure, M. Ralser, E. Blanco, L. Morey, M. Sansó, M. G. S. Palayret, B. Lehner, L. Di Croce, A. Wutz, B. Hendrich, D. Klenerman, and E. D. Laue. 3D structures of individual mammalian genomes studied by single-cell Hi-C. *Nature*, 544(7648):59–64, 6 Apr. 2017.

42. N. Varoquaux, I. Liachko, F. Ay, J. N. Burton, J. Shendure, M. J. Dunham, J.-P. Vert, and W. S. Noble. Accurate identification of centromere locations in yeast genomes using Hi-C. *Nucleic Acids Res.*, 43(11):5331–5339, 23 June 2015.

43. H. Wong, H. Marie-Nelly, S. Herbert, P. Carrivain, H. Blanc, R. Koszul, E. Fabre, and C. Zimmer. A predictive computational model of the dynamic 3D interphase yeast nucleus. *Curr. Biol.*, 22(20):1881–1890, 23 Oct. 2012.
