# Supplementary material for: Sim3C: simulation of Hi-C and Meta3C proximity ligation sequencing technologies
Source: Gigascience. 2017 Nov 15;7(2):1–12. doi: 10.1093/gigascience/gix103 (PMC5827349; doi:10.1093/gigascience/gix103)
Supplement: Response-to-Reviewer-Comments_Original-Submission.pdf [file gix103_response-to-reviewer-comments_original-submission.pdf]

Author's Response To Reviewer Comments

Please note that we have attached a cover letter and a more easily read .doc file of our responses which we have included here.

Responses to Reviewer #1 feedback:

1. Overly long

The manuscript has been shortened in the initial description and a figure of limited value (empirical distribution) has been removed.

2. Goals of simulator are not clear

The introduction has been revised to make the goal/motivations for developing the tool more clear.

3. Details obfuscate main goal

Clarification of goals was added to the introduction.

4. Too many figures, condense.

We have removed figure 2 as it was of limited value. The three following figures (3,4,5) have been combined into one.
We do not wish to combine heatmap figures any further as they would lose their detail if further shrunk and combine too many concerns into a single caption.

5. Real data sets exist

Availability of real data will always be limited and the precision of a priori knowledge even more so. Simulation meanwhile can offer explicit control over and exact knowledge of data characteristics, which can be crucial for algorithm development. Not just in solving the initial problem effectively, but also knowing when we have failed and where we might likely fail. We currently accept “best we can do” when testing or we avoid approaches entirely because of lack of sufficient a priori data or unavailability of precise enough, finely grained enough real data series.

6. Are all replicons treated as circular

Community replicons can be treated as either wholly circular or linear. We have a working branch where a more expressive community definition is possible. Due to the unavoidable -- though hopefully slight -- increased complexity, our intention is to take greater advantage of this change prior to merging back with the main branch. In particular, an additional goal would be modelling externally determined structural details.

7. TADs, how are they decided upon

TADs are treated as being drawn uniformly random. The option to supply locations has been considered for future versions. Our present intention with random modelling of smaller structural features is to guard against the possibility that, were they entirely absent, it might result in data that is so simplistic that algorithms to analyse metagenomic Hi-C data might perform unrealistically well. Further work is required (and on-going) to allow users to define regions of interaction. When done we hope this would include structures such as centromeres.

8. Combine figure 1 and 2

Figure 2 has been removed and figures 3, 4 and 5 have been combined.

Fig 8. No description for C and D
The caption for this figure was inadvertently replaced with the wrong text during the final draft process. This has now been corrected.

Responses to Reviewer #2 feedback:

1. Provide more explanation and technical details

The revised manuscript attempts to improve the clarity of description without adding length, in order to strike a balance between this request for added detail and the previous reviewer's concern that it was already too long.

2. Fig 3. Log scale x and y.

Taking feedback of both reviewers, we have elected instead to remove this figure (of limited value) and condense the manuscript.

3. Add chromatin loops as a simulation choice?

The main focus of our own work has been microbial communities, and as a consequence the simulation of features seen in large multicellular organisms, although of much merit, has taken lower priority. As it stands, the simulator cannot reproduce such structural details as seen in eukaryotic genomes. We do wish to provide this in future, however.

4. Is it reproducible? How does it compare to real replicates

Sim3C makes consistent use of random seeds throughout the simulation, therefore run to run using the same runtime parameters, we would expect there to be no variation. Were a user to vary only the seed value to produce replicates, we would expect run to run variation to be much less than real replicates, particularly when simulating the simplest model, with no TAD approximation. Primarily, this is because Sim3C treats experimental parameters offered on the

command-line as exact values and does not apply any type of noise. More run to run variation could be introduced by varying runtime parameters besides the seed. This is something we regard as outside of the core simulation and instead as one of the many user-driven use cases that Sim3C can support. Also, due to their random generation, if TAD approximation is enabled, variation would of course be larger but run to run simulations would now represent systematically different chromosomal folding.

5. Human and mouse data sims?

Were Sim3C able to model structural details present only in these genomes or offer models driven by externally determined observations (motif detection, experimentally determined coordinates) then we would also agree in the necessity of including such genomes as human or mouse. As it stands, the present Sim3C feature set is effectively demonstrated with yeast as the most complex single genome.

6. Hi-C vs HiC, sciHiC vs sciHi-C

The terms have been changed to Hi-C and sciHi-C. Our original motivation to omit hyphens in these short labels was in consideration of the treatment of hyphens within search indexing.
