# Supplementary material for: Sim3C: simulation of Hi-C and Meta3C proximity ligation sequencing technologies
Source: Gigascience. 2017 Nov 15;7(2):1–12. doi: 10.1093/gigascience/gix103 (PMC5827349; doi:10.1093/gigascience/gix103)
Supplement: Response-to-Reviewer-Comments_Revision-1.pdf [file gix103_response-to-reviewer-comments_revision-1.pdf]

Author's Response To Reviewer Comments

We have made the requested minor revisions.
