# Supplementary material for: Sim3C: simulation of Hi-C and Meta3C proximity ligation sequencing technologies
Source: Gigascience. 2017 Nov 15;7(2):1–12. doi: 10.1093/gigascience/gix103 (PMC5827349; doi:10.1093/gigascience/gix103)
Supplement: Reviewer-1-Report-(Original-Submission).pdf [file gix103_reviewer-1-report-(original-submission).pdf]

Reviewer Report

Title: Sim3C: Simulation Of HiC And Meta3C Proximity Ligation Sequencing Technologies

Version: Original Submission **Date:** 6/12/2017

Reviewer name: Andrew Adey

Reviewer Comments to Author:

In the manuscript by DeMaere and Darling, the authors describe their computational simulator for HiC and 3C sequencing that models the 3D arrangement of chromatin and how that arrangement is conveyed via proximity ligation methods. Overall the manuscript is long and does not clearly describe the main goals of the simulator. The detail is appreciated, but not when it obfuscates the main goal of the manuscript. Also the figures could be condensed so that there are less figures with more panels. That being said, I do believe the simulator that the authors have developed is very sophisticated and appears to work well with a few exceptions. The major issue is the packaging of the method into more a concise and clear text. Below are some more specific comments:My first thought is regarding where this simulator will be particularly useful? The authors mention it is primarily for software tool development and that the cost of generating HiC/3C data is very high and that many of the existing datasets are sparse. However, there are many existing datasets that are extremely rich and deep that would seem more appropriate. While I am not convinced on the utility for software development when abundant real data is publicly available, I do agree that having means to simulate sequence read data may have other valuable applications - primarily in exploring power in deconvolving metagenomic samples.For the eukaryotic simulated data there is a clear stretch of signal this is perpendicular to the diagonal as is typically observed for circular genomes, though this would not be expected for linear chromosomes (e.g. Figure 7). Does the simulator assume all chromosomes are circular? This is odd and needs to be addressed.Also on figure 7, the authors are highlighting that there is a greater inter chromosomal signal when compared to real data - is that a good thing? I can see that it may be desirable if the goal is to generate signal that would be generated under the assumption that there is no chromatin organization in the genome and thus be used as a background model. I can see this as a potential use, but it should be more clearly stated.The authors describe the ability to simulate TADs - however it is not clearly described how the TADs are decided upon - can users specify where TADs should be located (e.g. if they have a callset of TADs and want to create data simulating them that they can then alter - e.g. change one TAD and see how it effects signal nearby so they can know what to expect for an experiment where they may be altering TAD-forming loci). Or are they only created randomly (which seems the case given page 8 line 212). This could also be more clearly described by stating broadly what is done then going into the methods of how that is accomplished.Figure 2 is an extremely simple and small diagram - could it not just be added into figure 1? It seems a bit excessive to stand as its own figure. This goes for several other figures.Figure 8 - there is no description for c and d panels. I assume c is real and d is simulated. The strong perpendicular band midway through the chromosome is observed which is discouraging as I have commented on for Figure 7.

Level of Interest

Please indicate how interesting you found the manuscript: An article whose findings are important to those with closely related research interests

Quality of Written English

Please indicate the quality of language in the manuscript: Not suitable for publication unless extensively edited

Declaration of Competing Interests

Please complete a declaration of competing interests, considering the following questions:

- Have you in the past five years received reimbursements, fees, funding, or salary from an organisation that may in any way gain or lose financially from the publication of this manuscript, either now or in the future?
- Do you hold any stocks or shares in an organisation that may in any way gain or lose financially from the publication of this manuscript, either now or in the future?
- Do you hold or are you currently applying for any patents relating to the content of the manuscript?
- Have you received reimbursements, fees, funding, or salary from an organization that holds or has applied for patents relating to the content of the manuscript?
- Do you have any other financial competing interests?
- Do you have any non-financial competing interests in relation to this paper?

If you can answer no to all of the above, write 'I declare that I have no competing interests' below. If your reply is yes to any, please give details below.

I declare that I have no competing interests.

I agree to the open peer review policy of the journal. I understand that my name will be included on my report to the authors and, if the manuscript is accepted for publication, my named report including any attachments I upload will be posted on the website along with the authors' responses. I agree for my report to be made available under an Open Access Creative Commons CC-BY license (http://creativecommons.org/licenses/by/4.0/). I understand that any comments which I do not wish to be included in my named report can be included as confidential comments to the editors, which will not be published.

I agree to the open peer review policy of the journal
