# Supplementary material for: Sim3C: simulation of Hi-C and Meta3C proximity ligation sequencing technologies
Source: Gigascience. 2017 Nov 15;7(2):1–12. doi: 10.1093/gigascience/gix103 (PMC5827349; doi:10.1093/gigascience/gix103)
Supplement: Reviewer-2-Report-(Original-Submission).pdf [file gix103_reviewer-2-report-(original-submission).pdf]

Reviewer Report

Title: Sim3C: Simulation Of HiC And Meta3C Proximity Ligation Sequencing Technologies

Version: Original Submission **Date:** 6/19/2017

Reviewer name: Ming Hu

Reviewer Comments to Author:

In this paper, the authors developed a software package Sim3C to simulate Hi-C data and other 3C-based data. This work addresses a very important research question, and has the potential to become a useful computational tool in genomics research. However, the authors need to provide more explanations and technical details to further improve the current manuscript. Here are my specific comments:Major comments:1. Figure 3. It is better to plot Figure 3 in log scale for both x-axis and y-axis. In log scale, the slope of contact probably has direct biophysical interpretation, as described by the first Hi-C paper (Lieberman-Aiden et al, Science, 2009). I am very curious to see how biophysics model contributes to the data generation mechanism.2. In Rao et al, Cell, 2014 paper, they identified chromatin loops anchored by CTCF motifs. In Sim3C, the authors considered the 1D genomic distance effect and hierarchical TAD structures. It would be great if Sim3C can also take chromatin loops into consideration.3. Hi-C data can help to detect allelic-specific chromatin interactions. Is Sim3C able to simulate allelic specific proximity ligation data?4. It is very important to rigorously evaluate the data reproducibility. Using Sim3C, if users simulate Hi-C data multiple times with different random seeds, would the reproducibility between two simulated datasets be comparable to the reproducibility between two real biological replicates?5. The authors showed simulated contact matrices of bacteria (Figure 6) and budding yeast (Figure 7). They also need to simulate both human and mouse genome-wide contact matrices, and compare the simulated contact matrices with real data.Minor comments:1. Please replace all 'HiC' by 'Hi-C'.2. Page 6, line 116, "sciHiC" should be "scHi-C".

Level of Interest

Please indicate how interesting you found the manuscript: An article whose findings are important to those with closely related research interests

Quality of Written English

Please indicate the quality of language in the manuscript: Acceptable

Declaration of Competing Interests

Please complete a declaration of competing interests, considering the following questions:

- Have you in the past five years received reimbursements, fees, funding, or salary from an organisation that may in any way gain or lose financially from the publication of this manuscript, either now or in the future?
- Do you hold any stocks or shares in an organisation that may in any way gain or lose financially from the publication of this manuscript, either now or in the future?
- Do you hold or are you currently applying for any patents relating to the content of the manuscript?
- Have you received reimbursements, fees, funding, or salary from an organization that holds or has applied for patents relating to the content of the manuscript?
- Do you have any other financial competing interests?
- Do you have any non-financial competing interests in relation to this paper?

If you can answer no to all of the above, write 'I declare that I have no competing interests' below. If your reply is yes to any, please give details below.

I declare that I have no competing interests

I agree to the open peer review policy of the journal. I understand that my name will be included on my report to the authors and, if the manuscript is accepted for publication, my named report including any attachments I upload will be posted on the website along with the authors' responses. I agree for my report to be made available under an Open Access Creative Commons CC-BY license (http://creativecommons.org/licenses/by/4.0/). I understand that any comments which I do not wish to be included in my named report can be included as confidential comments to the editors, which will not be published.

I agree to the open peer review policy of the journal
